# Biological and Clinical Factors contributing to the Metabolic Heterogeneity of Hospitalized Patients with and without COVID-19

**DOI:** 10.21203/rs.3.rs-480167/v1

**Published:** 2021-05-10

**Authors:** Angelo D’Alessandro, Imo Akpan, Tiffany Thomas, Julie Reisz, Francesca Cendali, Fabia Gamboni, Travis Nemkov, Kiruphagaran Thangaraju, Upendra Katneni, Kenichi Tanaka, Stacie Kahn, Alexander Wei, Jacob Valk, Krystalyn Hudson, David Roh, Chiara Moriconi, James Zimring, Eldad Hod, Steven Spitalnik, Paul Buehler, Richard Francis

**Affiliations:** University of Colorado Denver; Columbia University Irving Medical Center; Columbia University; University of Colorado Denver; University of Colorado Denver; University of Colorado Denver; Department of Biochemistry and Molecular Genetics, University of Colorado Denver; University of Maryland; University of Maryland; University of Maryland; Columbia University Irving Medical Center; Columbia University Irving Medical Center; Columbia University Irving Medical Center; Columbia University Medical Center; Columbia University; Columbia University Irving Medical Center; University of Virginia; Columbia University; Columbia University Irving Medical Center; University of Maryland; Columbia University Irving Medical Center

**Keywords:** COVID-19, plasma metabolome, acute critical illnesses

## Abstract

The Corona Virus Disease 2019 (COVID-19) pandemic represents an ongoing worldwide challenge. Exploratory studies evaluating the impact of COVID-19 infection on the plasma metabolome have been performed, often with small numbers of patients, and with or without relevant control data; however, determining the impact of biological and clinical variables remains critical to understanding potential markers of disease severity and progression. The present large study, including relevant controls, sought to understand independent and overlapping metabolic features of samples from acutely ill patients (n = 831), testing positive (n = 543) or negative (n = 288) for COVID-19. High-throughput metabolomics analyses were complemented with antigen and enzymatic activity assays on 831 plasma samples from acutely ill patients while in the emergency department, at admission, and during hospitalization. We then performed additional lipidomics analyses of the 60 subjects with the lowest and highest body mass index, either COVID-19 positive or negative. Omics data were correlated to detailed data on patient characteristics and clinical laboratory assays measuring coagulation, hematology and chemistry analytes. Significant changes in arginine/proline/citrulline, tryptophan/indole/kynurenine, fatty acid and acyl-carnitine metabolism emerged as highly relevant markers of disease severity, progression and prognosis as a function of biological and clinical variables in these patients. Further, machine learning models were trained by entering all metabolomics and clinical data from half of the COVID-19 patient cohort and then tested on the other half yielding ~ 78% prediction accuracy. Finally, the extensive amount of information accumulated in this large, prospective, observational study provides a foundation for follow-up mechanistic studies and data sharing opportunities, which will advance our understanding of the characteristics of the plasma metabolism in COVID-19 and other acute critical illnesses.

## Introduction

On April 28 2020 we began performing one of the earliest investigations on the impact of SARS-CoV-2 infection on the circulating metabolome.^1^ At that time, we reported that ~ 3 million cases had been confirmed worldwide, a number that has dramatically risen since to ~ 140 million cases and 3 million deaths – according to the World Health Organization (https://www.who.int/emergencies/diseases/novel-coronavirus-2019). Our original study aimed to identify metabolic signatures that could help inform prognosis and guide treatment early after the onset of COVID-19, the disease caused by SARS-CoV-2 infection. Indeed, small molecule metabolites provide the building blocks that fuel viral replication, from nucleic acids to proteins and membrane lipids. SARS-CoV-2^1–5^ – like other viral infections^6^ – was found to promote the mobilization of free fatty acids to support the formation of viral capsid-associated membranes, a phenomenon that could be explained, at least in part, by activation of phospholipase A2,^7,8^ a target amenable to pharmacological intervention.

Despite public health interventions and the advent of multiple vaccination strategies, the number of cases in the United States has plateaued, rather than continuing to decrease, and COVID-19 cases continue to rise globally. This may be explained by multiple factors, including (i) vaccination rates lagging behind the percentage required to reach herd immunity; (ii) reopening too early, while discontinuing public health mandates; and (iii) the emergence of variants with more efficient transmission,^9,10^ which may also escape acquired immunity and/or vaccination.^11,12^ Therefore, efforts aimed at identifying strategies to treat SARS-CoV-2, including metabolic interventions or repurposing drugs with potential metabolic targets,^13^ remain important.

To this end, herein we collected a large independent data set, including patient characteristics, clinical information, and routine and specialized clinical laboratory results of acutely ill patients, who tested either positive or negative for SARS-CoV-2. This large patient group allowed for verification and expansion of previously published metabolomics studies in COVID-19 and other acute critical illnesses. Our 2020 study,^1^ like most subsequent studies,^2–5,14–21^ evaluated small cohorts of COVID-19 patients, with varying severity of disease, and often used healthy individuals as controls. Therefore, some of the reported observations could result from inflammation or infection, in general, rather than COVID-19, in particular. For example, activation of the kynurenine pathway^1,16,20,22,23^ could result from inflammatory or interferon-activated responses to viral infection,^24^ and from downstream activation of indole 2,3-dioxygenase. Based on these observational reports, it was correctly^25^ predicted that subjects with basal activation of these pathways (e.g., Down syndrome) may be more susceptible to life-threatening COVID-19.^1,26^

The published metabolomics studies on COVID-19 were not powered to characterize the effects of other variables critical for disease severity and prognosis. As exsmples, evaluation of biological (e.g., sex,^27^ age,^28^ ethnicity,^29^ body mass index,^30^ blood group^31^) and clinical (e.g., obesity, diabetes, cardiovascular disease, kidney disease)^32^ characteristics are necessary to define independent and overlapping metabolic findings in COVID-19 and other acute diseases. To this end, in some cases, we performed sub-analyses focusing on one variable at a time, like sex^3,23^ or inflammation (e.g., circulating interleukin-6 (IL-6) levels).^1,4^

Another limitation of previous studies, including ours,^1^ was the lack of longitudinal data to evaluate progression of metabolic dysfunction in hospitalized patients with severe COVID-19 or other acute illnesses. Nonetheless, some studies tried addressing this,^4,17^ and identified distinct omics phases corresponding to the acute response to infection (Stage 1) and then the development of antibody responses (Stage 2).^33^ Herein, we expand this approach, as a proof-of-principle, by comparing three mechanically-ventilated COVID-19 patients at up to 21 longitudinal time points; these data suggest a pattern of metabolic changes with relevance to prognosis in severe COVID-19 cases.

## Results

### COVID-19 patients display significant markers of kidney injury, including increases in creatinine and purine oxidation, and decreases in amino acids

**Figure 1:**
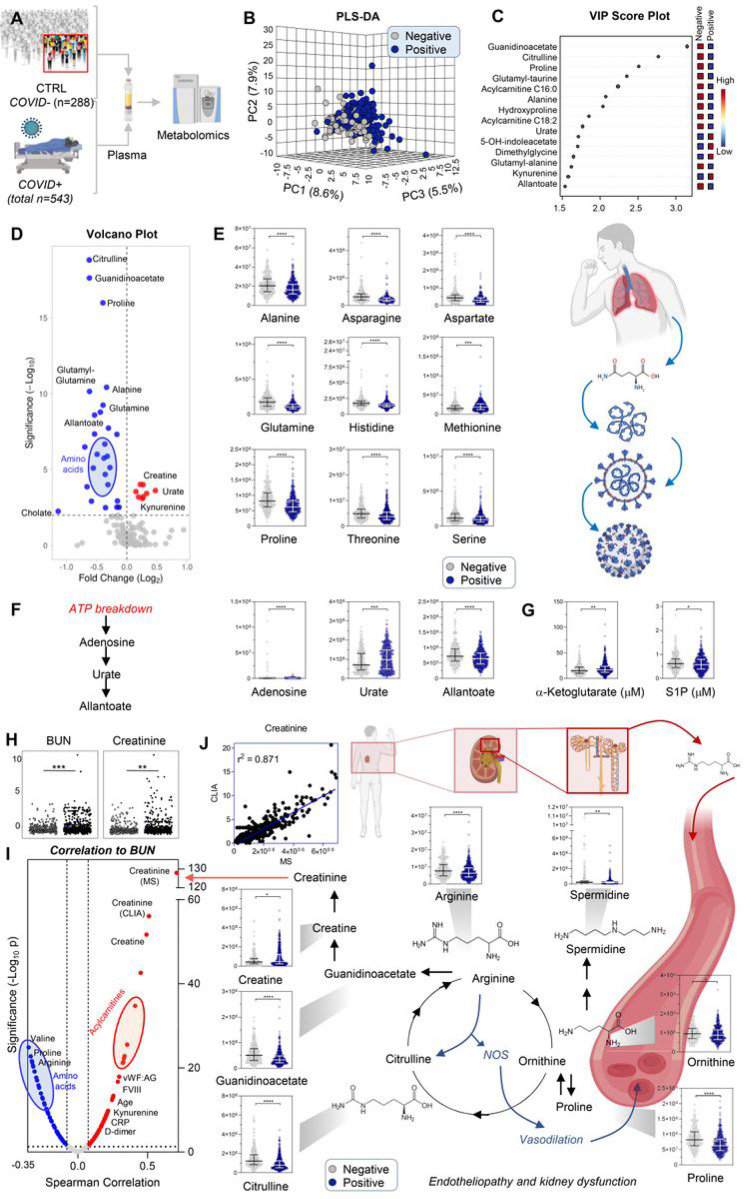
Metabolomics of hospitalized patients with (n=543) and without (n=288) COVID-19 (A). Partial least square-discriminant analysis of metabolomics data separated the two cohorts (B). Top 15 metabolites with the highest loading weights are indicated in the variable importance in projection (VIP) ranked list in C. In D, the volcano plot highlights significant effects of COVID-19 on plasma amino acid levels and purine oxidation. Violin plots (including median + ranges) are shown for amino acids (E) and purines (F) from relative quantitative analyses, and for two markers of mitochondrial dysfunction and hypoxia, alpha-ketoglutarate and sphingosine 1-phosphate (S1P) using absolute quantitative analyses against stable isotope-labeled internal standards in G. In H, blood urea nitrogen (BUN) and creatinine, markers of kidney dysfunction, were significantly increased in COVID-19 patients. Metabolic and clinical correlates of BUN (top positive correlate being creatinine) are in I. A significant positive correlation (p<0.0001; r2 = 0.871) was observed between creatinine measurements via CLIA-certified and mass spectrometry (MS)-based approaches (J). In J, violin plots highlight metabolites in the arginine, proline, and creatine metabolism.

**Figure 2:**
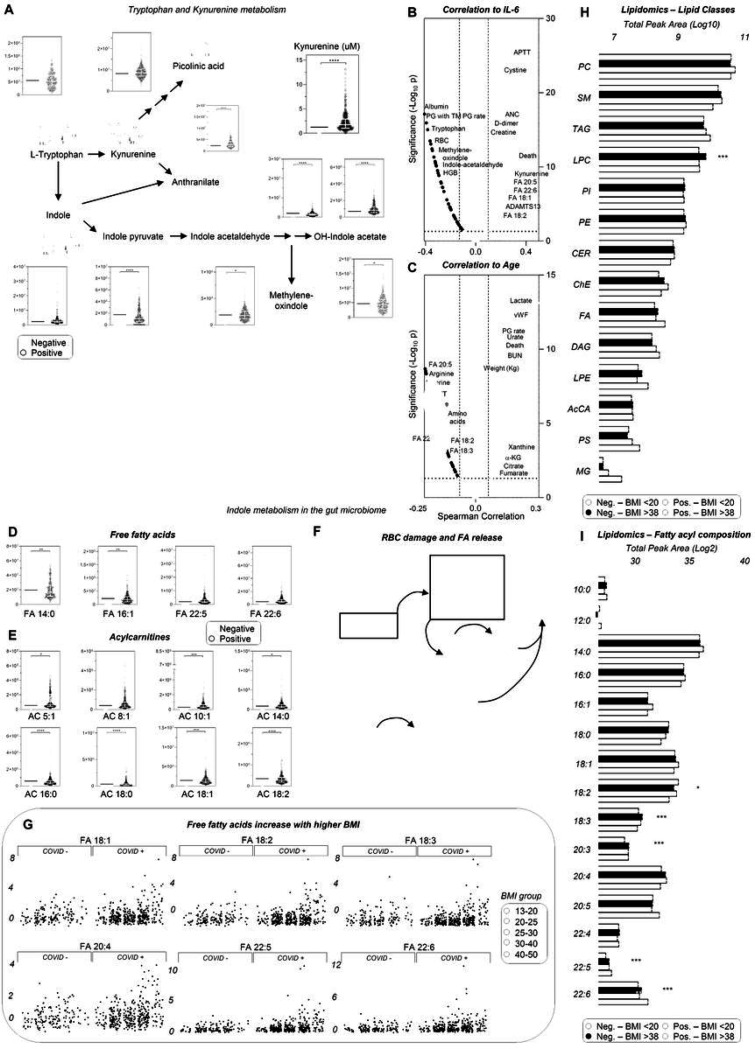
Alteration of tryptophan/kynurenine/indole metabolism as a function of inflammation, and dysregulation of lipid metabolism as a function of body mass index in hospitalized patients with and without COVID-19. In A, violin plot of tryptophan metabolism as a function of COVID status (median + range). Metabolic and clinical correlates to interleukin 6 (IL-6) as a marker of inflammation (B) and patient age (C) indicate a strong correlation of this pathway and lipid metabolism, especially free fatty acids (D) and acyl-carnitines (E), with disease state. Free fatty acids may derive from blood cell vesiculation and/or mobilization from brown adipose tissue, a process that could fuel viral membrane formation (F). In G, breakdown of free fatty acid levels as a function of patients’ body mass index (BMI) and COVID-19 status. Lipidomics analyses of COVID-19-positive and -negative patients with BMI lower than 20 or higher than 38 revealed a significant impact of these variables on lipid class (H) and fatty acyl (I) composition.

**Figure 3:**
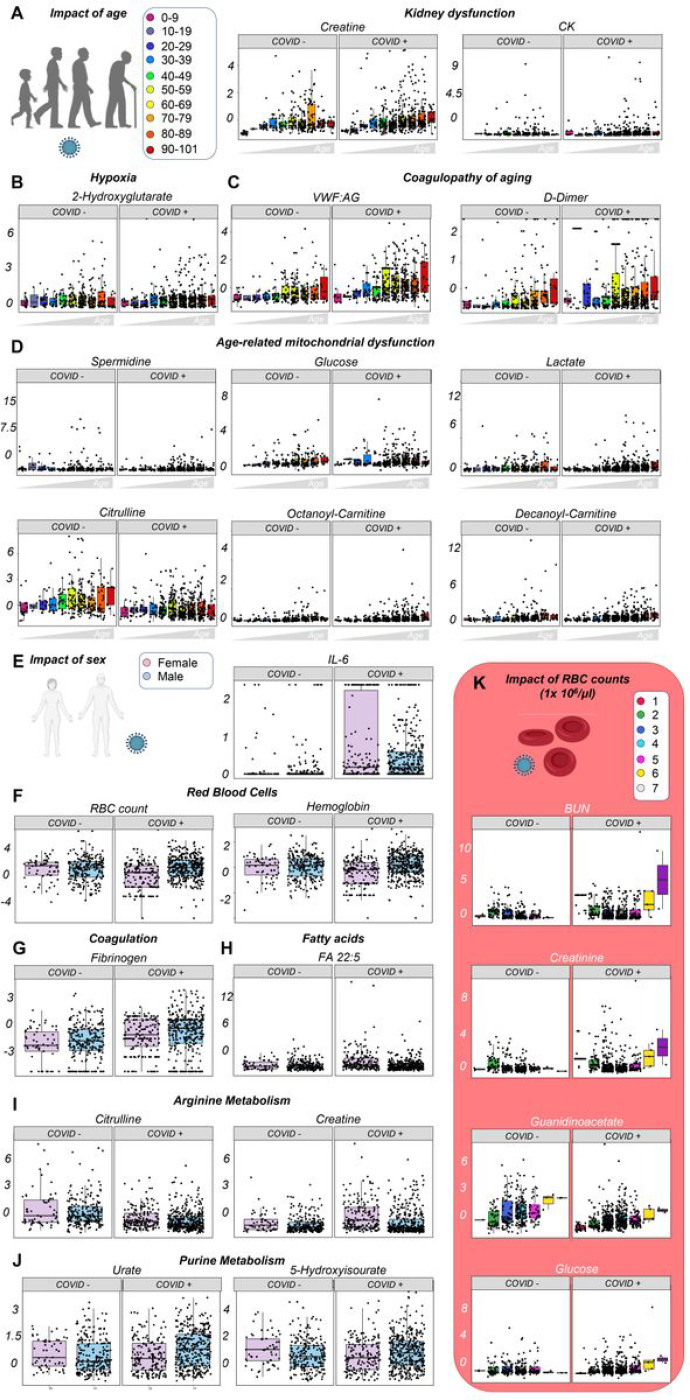
The impact of age and sex on the plasma metabolome of hospitalized patients with or without COVID-19. Patients were clustered into groups depending on their age (A). Significant correlates to age or COVID-19 status were identified through Spearman correlation and two-way ANOVA, with top variables including markers of kidney dysfunction (A), hypoxia (B), coagulopathy (C), and age-related mitochondrial dysfunction (D). Similar analyses were performed as a function of patients’ COVID-19 status and sex (E), with inflammatory markers being significantly affected by COVID-19, and RBC (F) and coagulation parameters (G) by sex. Similarly, sex affected fatty acid levels (especially poly- and highly-unsaturated, long chain fatty acids), and arginine and purine metabolism (H-J). Because of the impact of sex on RBC-related parameters, additional analyses were performed highlighting correlates to RBC counts and COVID-19 status, demonstrating a strong correlation with kidney dysfunction (K). All the metabolites shown in this figure as dot plots are significant by two-way ANOVA (FDR < 0.05).

**Figure 4:**
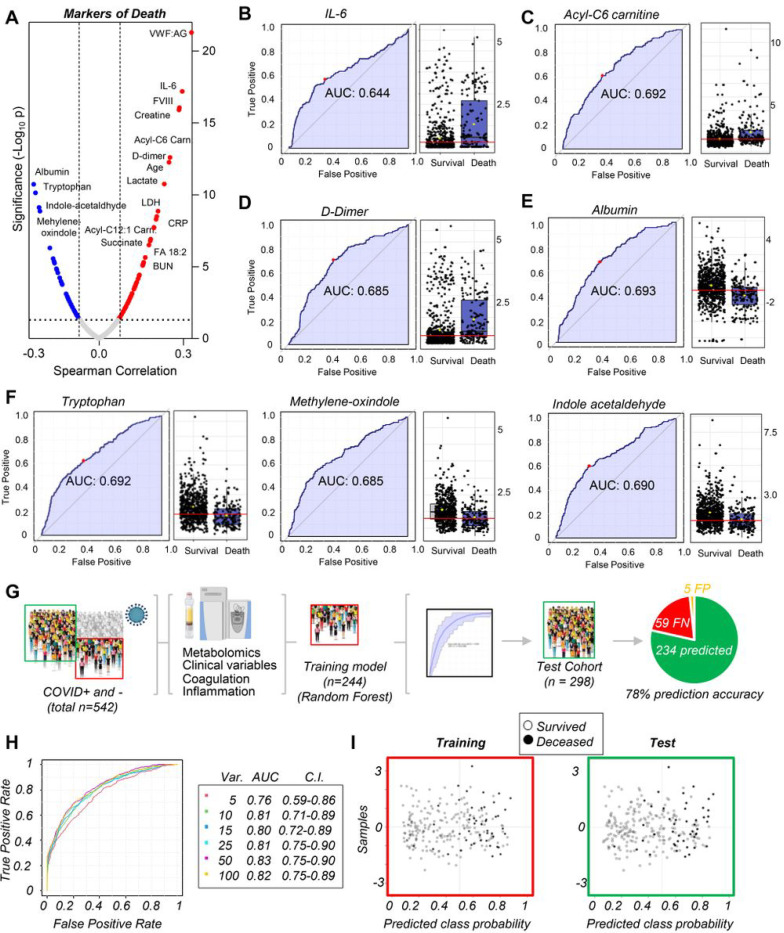
Markers of mortality in hospitalized patients, including COVID-19 patients. In A, clinical and metabolic markers of mortality were ranked from Spearman correlation analyses (y axes indicate −log10 of p-values). Because mortality is a non-continuous variable, additional univariate (B-F) and multivariate (G) biomarker analyses were performed to calculate ROC curves and train machine learning algorithms (random forest in this figure, supporting vector machine in the Supplement) to predict mortality in hospitalized COVID-19 patients based on the top 10 clinical and metabolic variables (H), a model that yielded 78% prediction accuracy (G-I).

**Figure 5:**
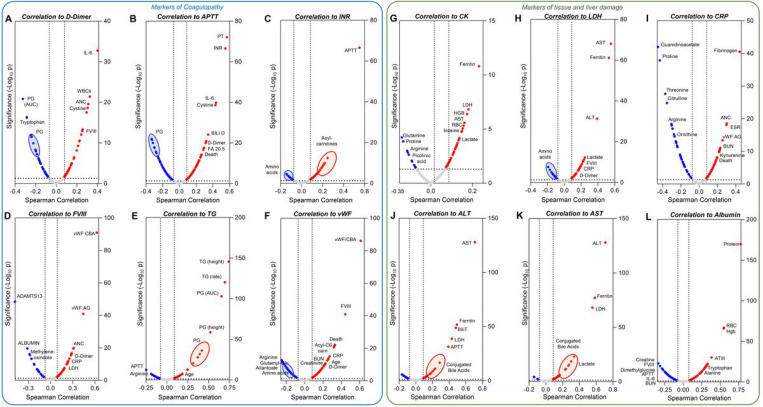
Metabolic correlates to coagulation parameters and markers of tissue and liver damage. Spearman correlation analyses correlated clinical and metabolic parameters to coagulation status (A-F) or tissue damage (G-L). Volcano plots represent metabolites that have significant (p<0.05) positive (red) or negative (blue) correlations with any of the parameters. Parameters are abbreviated using standard clinical terms.

**Figure 6:**
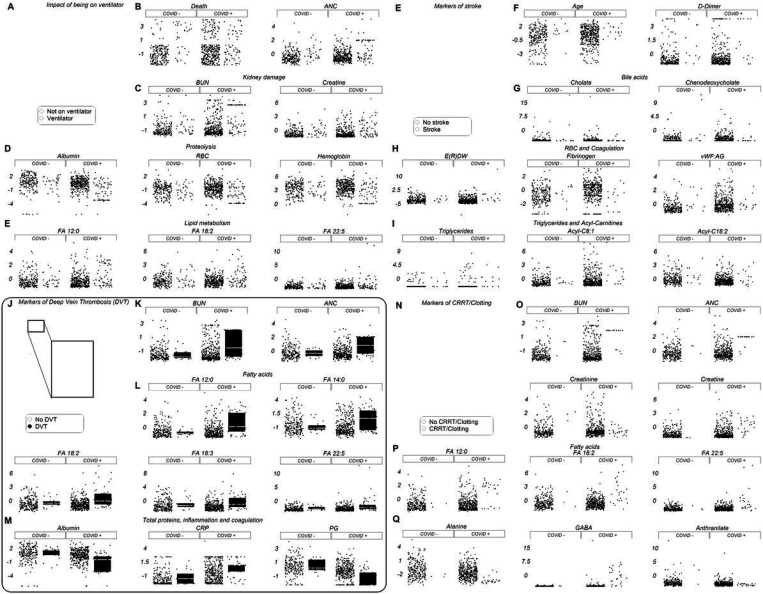
Clinical complications and metabolic/clinical markers. Hospitalized patients, with and without COVID-19, were divided into subgroups depending on clinical complications (e.g., stroke, deep vein thrombosis) and/or interventions (e.g., ventilators, hemodialysis). All metabolites shown in this figure as dot plots are significant by two-way ANOVA (FDR<0.05).

**Figure 7:**
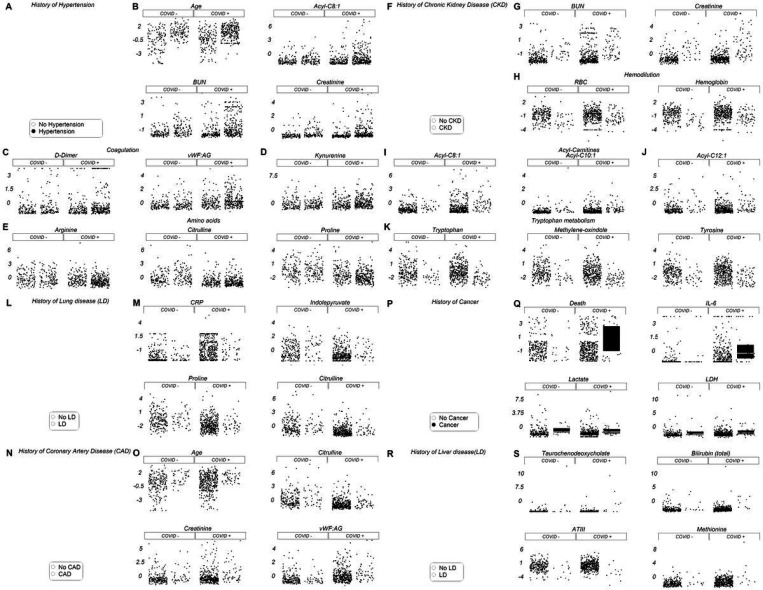
Pre-existing conditions and metabolic/clinical markers. Hospitalized patients, with and without COVID-19, were divided into subgroups depending on clinical history. Specifically, patients were identified who presented with a history of hypertension (A-E), chronic kidney disease (F-K), lung disease (L-M), coronary artery disease (N-O), cancer (P-Q), or liver disease (R-S). All metabolite/clinical variables shown in this figure as dot plots are significant by two-way ANOVA (FDR<0.05).

**Figure 8:**
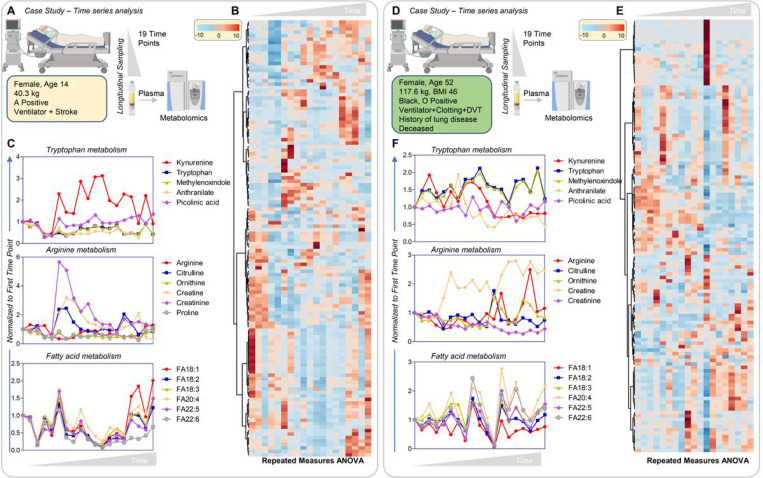
Time-course analysis of two patients with severe COVID-19, one surviving (A-C) and one dying (D-F) of disease. Both patients were ventilatated with coagulopathic complications, either stroke or deep vein thrombosis. The first patient, a 14-year old female with no pre-existing conditions, survived at the end of the time course and manifested transient activation of the kynurenine pathway and accumulation of creatinine (kidney dysfunction), which resolved early (C). This patient was also characterized by late accumulation of plasma free fatty acids (18C, 20C, and 22C poly- and highly-unsaturated fatty acids). The second patient, a 52-year old female with a history of obesity and lung disease, did not survive COVID-19; no activation of the kynuenine pathway was observed and creatine levels remained elevated.

Metabolomics analyses performed for 543 samples from COVID-19-negative patients were compared with those performed for 288 samples from acutely ill COVID-19-negative patients (Fig. 1.A). Raw data, along with clinical characteristics are detailed in Supplementary Table 1; a visual representation of technical mixes as a quality control against test samples confirmed good reproducibility (CV < 20%) in the tSNE analysis (Supplementary Fig. 1). Our prior studies^1^ compared the metabolome of COVID-19 patients to healthy controls; this prevented defining a metabolic signature specific to COVID-19 that could be differentiated from other acute illnesses or infections. Therefore, here we enrolled hospitalized patients who were COVID-19-negative (by PCR and/or serology), some of whom had non-COVID-19 respiratory tract infections. Not surprisingly, the metabolic phenotypes of these COVID-19-negative patients partially overlapped with those who were COVID-19-positive (Fig. 1.B). Figure 1.C shows which metabolites differentiate between these two groups, using variable importance in projection (VIP) scores in the PLS-DA analysis. Volcano plot elaborations (Fig. 1.D) also clearly showed decreased levels of almost all amino acids (Fig. 1.E) and increased levels of purine oxidation products (Fig. 1.F) in COVID-19 patients. Several of these observations were validated using stable isotope-labeled internal standards for absolute quantification (Supplementary Table 1), including markers of hypoxia,^34,35^ the carboxylic acid alpha-ketoglutarate, and sphingosine 1-phosphate (S1P) (Fig. 1.G). Notably, amino acid reabsorption occurs in the kidney^36^ and alterations in purine metabolism^37^ and S1P^38^ were recently tied to kidney ischemia and chronic kidney disease, respectively. In addition, moderate-severe kidney dysfunction was observed in all COVID-19 (+) patients, indicated by blood urea nitrogen (BUN) and creatinine levels (Fig. 1.H). The positive correlation between BUN and creatinine was paralleled by similar trends for acyl-carnitines (markers of kidney dysfunction^39^), and negative correlations between BUN and amino acids. As an internal validation of this approach, creatinine measured in the same samples by a CLIA-certified clinical chemistry assay and mass spectrometry correlated extremely well (p < 0.0001; r^2^ = 0.871 Spearman; Fig. 1.J). Overall, these results demonstrate significant up-regulation of creatine metabolism, accompanied by dysregulation of arginine catabolism to proline, polyamines, and citrulline (Fig. 1.J); also a hallmark of COVID-19-induced endotheliopathy.^40^ Interestingly, other markers of endothelial coagulopathy were also significantly increased in COVID-19 patients (Figs. 1–8), including VWF and its collagen binding activity (p< 0.0001). However, no significant differences in ADAMTS-13 levels or activity were observed; thus, VWF antigen:ADAMTS13 activity ratios were increased (p < 0.0001) favoring high molecular weight VWF oligomers and increased thrombotic potential.

### Up-regulation of the kynurenine pathway, due to inflammation, is inversely related to indole metabolism

Despite widespread decreases in most amino acids, circulating levels of kynurenine, a tryptophan catabolite (and other pathway intermediates) were confirmed^1,4,16,20,22^ to be significantly increased in COVID-19 patients as a function of IL-6 levels (Fig. 2.A–B). In contrast, indole metabolites, derived from tryptophan metabolism by the gut microbiome, were significantly decreased in COVID-19 patients (Fig. 2.A). Indeed, plasma levels of tryptophan/indoles and kynurenine were among the top negative and positive correlates with IL-6 levels (Fig. 2.B, Supplementary Fig. 2). IL-6 levels also positively correlated with coagulopathy markers (APTT, D-dimer), and mortality (Fig. 2.B). Positive correlations with age (Fig. 2.C) were observed for mortality and hypoxia markers,^41^ including lactate, purine oxidation products (xanthine, urate), markers of mitochondrial dysfunction (carboxylic acids citrate, alpha-ketoglutarate, succinate, fumarate), with a role in inflammation and thermogenesis via lipid catabolism.^42–44^ Consistently, COVID-19 induced increases in levels of long-chain poly- and highly-unsaturated free fatty acids and decreases in acyl-carnitines, although not saturated short and medium-chain ones (Fig. 2.D–E). Poly- and highly-unsaturated fatty acids positively correlated with IL-6 (Fig. 2.B) and negatively correlated with age (Fig. 2.C), but correlated positively with markers of kidney dysfunction (BUN), coagulation (vWF levels, plasmin generation - PG rate), and body weight. These results suggest increased lipid mobilization resulting from SARS-CoV-2-induced blood cell membrane vesiculation/lipolysis, as reported,^45^ and/or adipose tissue lipid catabolism, perhaps as a strategy for assembling viral membranes^6^ (Fig. 2.F). Given the role of obesity in COVID-19 outcomes,^46^ we evaluated metabolomics data in five body mass index (BMI) ranges, from underweight (BMI 13–20) to severely obese (BMI up to 50), highlighting a positive correlation between BMI and several 18, 20 and 22C series mono- and poly-unsaturated fatty acids in COVID-19-positive, as compared to COVID-19-negative patients (significant metabolites shown in Fig. 2.G, Supplementary Fig. 3). Therefore, lipidomics analyses were performed as a function of the lowest (< 20) and highest (> 38) BMI ranges (n = 15 subjects per group) and results separated by lipid class and fatty acyl-chain composition (Fig. 2.H–I; Supplementary Table 1). COVID-19 patients, especially those with highest BMI, had significantly higher levels of phosphatidylcholines (PCs), triacylglycerols (TAG), diacylglycerols (DAG), monoacylglycerols (MG), lysophosphatidylethanolamines (LPEs), and phosphatidylserines (PS; Fig. 2.H); these were particularly enriched in very long chain, highly-unsaturated fatty acids (20:3, 20:5, 22:5, 22:6) and depleted in 18C series fatty acids (stearic, oleic, linoleic) (Fig. 2.I).

### Effects of sex, age, and ethnicity on the plasma metabolome of hospitalized COVID-19 patients

Because older male COVID-19 patients have a poorer prognosis, as compared to young females,^3,23,46^ and given the large size of our cohorts, we tried identifying metabolic and clinical correlates for these variables (Fig. 3.A–D, Supplementary Fig. 4.E–J, Supplementary Fig. 5). Aging was associated with increased weight, BMI, kidney dysfunction (creatine, creatinine), and tissue damage (creatine kinase), along with markers of hypercoagulability (VWF:AG, FVIII), fibrinolysis (D-dimer), hyperglycemia, hypoxia and mitochondrial dysfunction (2-hydroxyglutarate, lactate, spermidine, acyl-carnitines), purine oxidation (urate), inflammation (CRP), proteolysis (albumin), and anemia (hemoglobin levels, RBC counts), especially in COVID-19 patients (Fig. 3.A–D).

Male patients, both with and without COVID-19, had higher RBC counts and hemoglobin levels, lower citrulline and creatine levels, lower levels of highly-unsaturated fatty acids (e.g., eicosapentaenoic, docosapentaenoic, docosahexaenoic acid; Fig. 3.E–I, Supplementary Fig. 5); however, only COVID-positive males, but not females, had increased urate levels (Fig. 3.J).

Because RBC count and hemoglobin level always strictly correlated (Supplementary Fig. 6) and were affected by both age and sex, we divided both cohorts into sub-groups based on RBC count (Fig. 3.K); this highlighted a positive correlation between RBC count and kidney damage (BUN, creatinine, guanidinoacetate), total protein level, and glycemia, along with negative correlations with one-carbon metabolites choline and methionine.

In these cohorts, race was also associated with inflammation, thromboinflammatory complications, body weight/BMI, and kidney dysfunction; indeed, IL-6, D-dimer, BUN, and creatinine levels were highest in individuals of African descent (Supplementary Fig. 7). In addition, plasma dimethylglycine, indole, and cystine levels were highest in individuals of African descent, whereas kynurenine levels increased in all COVID-19 patients independent of race. Interestingly, ABO blood group status, which is controversially associated with COVID-19 prognosis,^31^ indicated that the highest kynurenine, GABA, dimethylglycine, and creatinine levels were in blood group O subjects (Supplementary Fig. 8). Although our sample size was limited for blood group A COVID-19 patients (n = 111 samples), they had the with highest IL-6 levels (Supplementary Fig. 8).

### Markers of mortality in acutely-ill hospitalized patients

While previous studies identified prognostic and disease severity markers in COVID-19 patients, they studied relatively few patients and did not include hospitalized COVID-19-negative patients as controls.^1, 16–18,20,21,33,47,48^ To visualize ranking correlates of mortality, we performed preliminary correlation analyses of both our cohorts (Fig. 4.A), confirming strong positive correlations between mortality and markers of inflammation, coagulopathy, kidney and tissue damage and hypoxia. Because death is a non-continuous variable, biomarker analyses were also performed to calculate ROC curves for metabolites and clinical covariates at admission that significantly associated with poor outcomes independent of cohort (Fig. 3.B–F), or divided into COVID-19 patients and controls (Supplementary Fig. 9). Several of the highest-ranking variables (Fig. 3) included IL-6, acyl-carnitines (especially hexanoyl-carnitine), D-dimers, albumin, and tryptophan metabolites.

Because metabolomics data and clinical variables were available for 542 COVID-19 samples, we used 244 randomly-selected samples to train a machine learning model to predict mortality in these patients (Fig. 4.G). Data on training, ROC curves from multivariate models, prediction accuracy, and the top 15 variables fed into the model are shown in Supplementary Fig. 10.A–B for elaboration with the random forest or SVM algorithm. Overall, the top 10 variables from the random forest algorithm (Fig. 4.H) showed an AUC of 0.81 (confidence interval 0.71–0.89), resulting in the highest predictive ability with the fewest variables. Using the remaining 298 samples as a test set correctly predicted survival or death of 234 patients, with only 5 false positives (i.e., predicted to die, but survived) and 59 false negatives (i.e., predicted to survive, but died), demonstrating a 78% accuracy of the model, with high specificity (>95%), but moderate sensitivity (< 70%).

### Metabolic and clinical correlates to markers of coagulopathy and tissue damage

Correlation analyses (Spearman) identified clinical and metabolic correlates to coagulation parameters, including D-dimer, APTT, INR (> 96% positive correlation with APTT, thus excluded from the volcano plot), FVIII, TG, VWF:AG, and VWF:Collagen binding activity (Fig. 5.A–F). IL-6, age, metabolites linked to oxidant stress and sulfur metabolism (cystine), acyl-carnitines (markers of mitochondrial dysfunction^49^ and platelet activation^50,51^), and tryptophan and its metabolites, were top correlates to the coagulation parameters (Fig. 5.A–F).

Similarly, correlating metabolites and clinical parameters to markers of tissue damage (CK, LDH), inflammation (CRP), liver damage (ALT, AST), and proteolysis/hemodilution (albumin) identified a strong interaction with arginine/proline metabolism, ferritin/hemoglobin/RBC counts, and tryptophan/kynurenine metabolism (Fig. 5.G–L). Interestingly, conjugated bile acids, well-established markers of liver inflammation^52^, were positively correlated with liver transaminases (Fig. 5.J–K).

### Clinical and metabolic correlates to clinical complications: ventilators, stroke, deep vein thrombosis (DVT,) and hemodialysis

Leveraging the manually-curated clinical records for the enrolled patients, we identified clinical and metabolic markers correlating with mechanical ventilation (Fig. 6.A–E; Supplementary Fig. 11), stroke (Fig. 6.F–J), DVT (Fig. 6.K–N), and hemodialysis (with or without coagulopathy (Fig. 6.O–R and Supplementary Fig. 12) in both COVID-19 patients and controls. In all cases, the top markers were related to kidney dysfunction (BUN, creatinine), proteolysis/hemodilution (albumin, RBC count, hemoglobin, fibrinogen), free fatty acids (dodecanoic, linoleic, linolenic, docosapentaenoic), acyl-carnitines, triglycerides, and amino acid metabolism (especially tryptophan, choline, and GABA). Trends observed in controls were more dramatic in COVID-19 patients presenting with similar manifestations.

### The effects of clinical history and pre-existing conditions on the metabolome and clinical phenotype of acutely-ill hospitalized patients

Pre-existing conditions, including obesity, cardiovascular disease, kidney disease, cancer, and diabetes, are all associated with poorer prognosis in COVID-19.^32^ Meta-analysis of our cohorts (Fig. 7.A–S, Supplementary Fig. 12) indicated that older subjects are more likely to present with a history of hypertension, coronary artery disease, and/or diabetes (Fig. 7.A, O; Supplementary Fig. 12.A–B). Hypertension, chronic kidney disease, lung disease, and coronary artery disease share altered tryptophan and arginine/proline/citrulline metabolism, trends exacerbated by COVID-19. Carnitine metabolism and aromatic amino acids were increased in patients with a history of kidney disease (Fig. 7.F–K), whereas cancer was accompanied by increased lactate (perhaps resulting from a Warburg phenotype; Fig. 7.Q). A history of liver disease was accompanied by increased levels of conjugated bile acids (e.g., taurochenodeoxycholate), total bilirubin, and methionine (Fig. 7.S). Finally, a history of diabetes was associated with increased lactate and lactoyl-glutathione levels, the latter a marker of glyoxylase damage (Supplementary Fig. 13).

### Longitudinal sampling in severe COVID-19 patients

Sampling at admission allowed us to collect longitudinal samples from some patients. As illustrative, thought-provoking examples, the results with three severe COVID-19 cases, only two of whom recovered, are presented here. Figure 8 (vectorial version in Supplementary Figs. 14–16, data in Supplementary Table 1) shows hierarchical clustering of metabolites as a function of time (19 time points for 2 patients and 21 for the third patient). These three patients were female, 14-, 45-, and 52-years old, of different ethnicity and BMI. Despite similar disease severity (e.g. all mechanically ventilated, with either stroke, clotting, or DVT manifestations), only the surviving patients manifested a spike in kynurenine levels throughout their course, which was not observed in the patient who died (Fig. 8.C, F). Increased creatine/creatinine eventually resolved in the surviving patients, but not in the patient who died. The surviving patients also manifested increased free fatty acid levels at the latest time points, especially poly and highly-unsaturated fatty acids of the 18, 20, and 22C series; in contrast, the non-surviving patient exhibited late accumulation of acyl-carnitines and amino acids which did not resolve (Supplementary Fig. 16).

## Discussion

The present study provides the most extensive metabolomics analysis of COVID-19 patients to date, including 831 samples at admission from hospitalized patients and 59 longitudinal samples from three case studies. These analyses used state-of-the-art high-throughput metabolomics approaches,^53,54^ which allow not only simultaneous discovery of novel markers, but also quantitative validation of previously identified correlates to inflammatory states, renal dysfunction, and mortality by using stable isotope-labeled internal standards. Importantly, these mass spectrometry-based results were comparable to quantitative measurements using CLIA-certified clinical assays of various metabolites, including creatinine, suggesting that implementing clinical metabolomics^55^ strategies in next-generation clinical chemistry laboratories may eventually become feasible. Leveraging the combination of large omics datasets from COVID-19 patients and controls with manually-curated clinical records, allowed identification of novel metabolic correlates to biological variables and patient characteristics; these confirmed and significantly enhanced previous efforts in this disease.^56^

For example, despite a positive correlation with weight and BMI, aging was accompanied by decreased circulating levels of several poly- and highly-unsaturated fatty acids, consistent with reported age-dependent declines in unsaturated fatty acids in healthy blood donors^57^ and fatty acid desaturase activity, with functional implication in hematopoiesis.^58^ Aging was also accompanied by increased markers of hypoxia (e.g., lactate, citrate, alpha-ketoglutarate, fumarate), indicative of progressive mitochondrial dysfunction.^59^ Given the role of these metabolites in immunometabolism,^42^ older patients also demonstrated increased inflammation, especially COVID-19 patients, accompanied by poorer outcomes. Similarly, purine catabolism and oxidation products (e.g., urate, xanthine), hallmarks of ischemic^44^ and hemorrhagic^41^ hypoxic organ damage, increased with age. Importantly, mitochondria activity, aging, and inflammation are all associated with hypercoagulabiity,^49^ harmonizing our observational results with the known increased incidence of thromboembolic complications in COVID-19.

In contrast, aging, especially in COVID-19 patients, was accompanied by altered levels of free fatty acids and acyl-carnitines. The former may fuel viral membrane synthesis, which may be sustained by lipid mobilization from adipose tissue and other sources, similar to observations in trauma patients^60^ and following the pathological vesiculation of RBC membranes.^45^ Because obesity also leads to poor outcomes in COVID-19, lipidomics analyses of 60 subjects with the highest and lowest BMIs allowed identification of obesity-related lipid signatures in COVID-19 patients. In particular, neutral lipids (MG, DAG, TAG) and phospholipids (PC and LPE) were mobilized; the latter may result from release of methyl-groups from LPCs to meet one carbon demands for viral nucleotide synthesis or repair of oxidant-induced isoaspartyl-damage^61^.

These metabolic observations were exacerbated in COVID-19 patients and were consistent with disease severity, as indicated by clinical records and clinical measurements of markers of inflammation (IL-6, CRP), coagulopathy (D-dimers, APTT, INR, FVIII, VWF:AG, VWF:collagen binding activity, VWF:ADAMTS-13 activity ratios, thrombin and plasmin generation), and renal dysfunction (BUN, creatinine). Metabolic correlates of these clinical parameters are provided in this study, as part of the efforts aimed at compiling an encyclopedic characterization of metabolism in health and disease. For example, we found strong negative correlations between kidney dysfunction and circulating amino acid levels, as possible indicators of decreased renal reabsorption^36,37,62^ and hemodilution. As another example, positive correlations between pro-inflammatory conjugated bile acids and liver transaminases support prior findings of mechanistic interactions of these metabolites with IL-1 beta and hepatic stress.^52^ Interestingly, these metabolites were also associated with coagulopathy in trauma/hemorrhagic shock,^63^ and with microbiome dysbiosis related to iron metabolism^64^, observations informing the correlations in our study between ferritin levels, acute phase response proteins (CRP), and conjugated bile acids.

Besides aging and inflammation, other factors are also associated with poor outcomes in COVID-19. For example, the expression levels of angiotensin converting enzyme 2 (ACE2) receptor in enterocytes modulate disease severity, in that viral entry into cells is mediated by pairing of ACE2 with the viral spike protein.^65^ Notably, we confirm^1,4^ that arginine/proline/citrulline metabolism is an important pathway affected by COVID-19, which not only depends on kidney function, but also on enterocytes.^66^ Furthermore, arginase to nitric oxide synthase activity may influence the pro-/anti-inflammatory state of gut resident macrophages.^67^ In addition, circulating levels of arginine pathway metabolites can be affected by RBC arginase activity^45^, which is in turn affected by oxidant stress and can contribute to COVID-19-induced endotheliopathy.^40^

Indole metabolites of microbial origin^68^ were also significantly decreased in COVID-19 patients, especially in those with the poorest outcomes. These decreases may result from tryptophan depletion as a function of kynurenine pathway activation in COVID-19,^1,16,22,23^ especially in older males. We confirmed that kynurenine levels correlated with SARS-CoV-2 infection, disease severity, and mortality. Indeed, IL-6 levels and kynurenine/tryptophan ratios were among the top predictors of mortality in COVID-19 patients, confirming previous targeted analyses^20^ of our larger, independent cohort. However, as activation of interferon responses appear necessary for eliciting adaptive immunity against COVID-19,^33^ it is interesting that, in our longitudinal blood collections of the COVID-19 patient who died, plasma kynurenine levels did not increase. In contrast, because some metabolites in the kynurenine pathway are neurotoxic (e.g., picolinic acid, quinolinic acid)^69^, uncontrolled activation of this pathway may contribute to some neurological comorbidities of COVID-19 (e.g., brain fog, weakness, fatigue). Interestingly, declines in tryptophan-derived *de novo* nicotinamide synthesis is associated with aging and inflammation,^70^ suggesting that nutritionally replenishing NAD reservoirs (e.g., nicotinamide riboside) may be therapeutic in facilitating recovery from severe COVID-19.^71^

Depleting tryptophan to promote kynurenine synthesis may also lead to serotonin depletion, a key component of platelet dense granules with a role in platelet activation.^72^ This is relevant given the importance of coagulopathy in COVID-19, with increased plasma levels of FVIII, D-dimers, and VWF (i.e., increased VWF:collagen binding activity, increased VWF:ADAMTS-13 activity ratio), which are among the top correlates of mortality in our cohort. In addition, inflammation negatively correlated with albumin levels, perhaps due to inflammation-induced proteolysis, agreeing with previous reports that albumin predicts all-cause and cardiovascular mortality in chronic kidney disease patients.^73^ Albumin strongly correlated with total protein and hemoglobin levels, which were also among the top correlates with kidney dysfunction, thereby strengthening the evidence supporting RBC contributions to kidney physiology.^38^ In contrast, no major effects of ABO blood group were noted in our cohort, except for a link to IL-6 levels (highest in blood group A, corroborating prior evidence relating to increased disease severity^31^). Not surprisingly, ABO blood group was also linked to patient ethnicity in our cohort, which correlated with increased inflammation (IL-6), D-dimers, creatinine, and cystine (oxidant stress) in individuals of African descent.

Finally, as a proof-of-principle, we entered admission data (clinical and metabolomics) into machine learning algorithms, randomly selecting approximately half of the COVID-19 patient cohort as a training set and the other half as a test set. The resulting model exhibited high specificity (>95%), but moderate sensitivity (~ 70%). The prediction accuracy of these models may be affected by clinical contributors to the metabolic heterogeneity of hospitalized patients, such as elements of their medical history. Nonetheless, we report here for the first time that metabolic phenotypes of COVID-19 patients were most extreme in patients presenting with a history of hypertension, chronic kidney disease, lung disease, cancer, coronary artery disease, or lung disease.

Taken together, the extensively detailed information in this large, prospective, observational study will support future mechanistic studies and data sharing opportunities to enhance understanding of the plasma metabolism in COVID-19 and other acute critical illnesses.

## Materials And Methods

### Patients

This study was approved by the Institutional Review Board of Columbia University Irving Medical Center (CUIMC) (Protocol Number AAAT0680). Data were obtained for patients who were either admitted to the hospital or seen in the Emergency Department from April 14, 2020 through May 31, 2020 (i.e., before the identification of and routine testing for novel variants in the USA), and were evaluated for SARS-CoV-2 by RT-PCR, serology. As part of routine care, hemostasis was evaluated on STAR Evolution and STAR Max analyzers (Diagnostica Stago, Parsippany, NJ), hematology testing by Sysmex XN900 (Lincolnshire, IL), and chemistry testing by Roche Cobas c502 (Indianapolis, IN). Laboratory values, including antithrombin (AT), prothrombin time (PT)/international normalized ratio (INR), activated partial thromboplastin time aPTT, fibrinogen, d-dimer, white blood cell count (WBC), absolute neutrophil count (ANC), absolute lymphocyte count (ALC), absolute monocyte count (AMC), hemoglobin, red blood cell count (RBC), RBC distribution width (RDW), reticulocyte count, platelet count, IL-6, lactate dehydrogenase (LDH), lactic acid, procalcitonin, troponin, blood urea nitrogen (BUN), creatinine, glucose, total-, direct-, and indirect bilirubin, aspartate amino transferase (AST), alanine amino transferase (ALT), albumin, total protein, ferritin, C-reactive protein (CRP), erythrocyte sedimentation rate (ESR), creatine kinase (CK), triglycerides, and blood type, were collected. Laboratory data were obtained from the Clinical Data Warehouse at CUIMC after approval from the Tripartite Request Assessment Committee. Clinical and demographic data, including name, medical record number (MRN), sex, date of birth, age, race, ethnicity, weight, body mass index, comorbidities (hypertension, diabetes mellitus, coronary artery disease, renal disease, hyperlipidemia, liver disease, lung disease), intubation/ventilator requirement, continuous veno-venous hemofiltration (CVVH) requirement, radiographically-confirmed thrombotic complications (deep vein thrombosis, pulmonary embolism, stroke), clotting of CVVH, hospitalization course (admission date, date of Emergency Department presentation, discharge date), mortality and date of death) were collected manually by reviewing the electronic medical record. Data were collected retrospectively for patients treated at two New York-Presbyterian Hospital campuses (CUIMC and The Allen hospital). Residual platelet poor plasma samples were collected for subsequent analyses.

Sample processing and metabolite extraction: Plasma samples were extracted via a modified Folch method (chloroform/methanol/water 8:4:3), which completely inactivates other coronaviruses, such as MERS-CoV.^74^ Briefly, 20 μL of plasma were diluted in 130 μl of LC-MS grade water, 600 μl of ice-cold chloroform/methanol (2:1) was added, and the samples vortexed for 10 seconds. Samples were then incubated at 4°C for 5 minutes, quickly vortexed (5 seconds), and centrifuged at 14,000 x *g* for 10 minutes at 4°C. The top (i.e., aqueous) phase was transferred to a new tube for metabolomics analysis.

### Ultra-High-Pressure Liquid Chromatography-Mass Spectrometry metabolomics and lipidomics

Analyses were performed using a Vanquish UHPLC coupled online to a Q Exactive mass spectrometer (Thermo Fisher, Bremen, Germany). Samples were analyzed using a 5 and 17 min gradient as described.^53,54,75^ Solvents were supplemented with 0.1 % formic acid for positive mode runs and 1 mM ammonium acetate for negative mode runs. MS acquisition, data analysis and elaboration was performed as described. ^53,54,75^

#### Metabolomics:

UHPLC-MS metabolomics analyses were performed as described in method^53,54,75^ and application papers,^1,76^ using a Vanquish UHPLC system coupled online to a high-resolution Q Exactive mass spectrometer (Thermo Fisher, Bremen, Germany). Samples were resolved over a Kinetex C18 column (2.1 ×150 mm, 1.7 μm; Phenomenex, Torrance, CA, USA) at 45°C. A volume of 10 ul of sample extracts was injected into the UHPLC-MS. Each sample was injected and run four times with two different chromatographic and MS conditions as follows: 1) using a 5 minute gradient at 450 μL/minute from 5–95% B (A: water/0.1% formic acid; B:acetonitrile/0.1% formic acid) and the MS was operated in positive mode and 2) using a 5 minute gradient at 450 μL/minute from 5–95% B (A: 5% acetonitrile, 95%water/1 mM ammonium acetate; B:95%acetonitrile/5% water, 1 mM ammonium acetate) and the MS was operated in negative ion mode. The UHPLC system was coupled online with a Q Exactive (Thermo, San Jose, CA, USA) scanning in Full MS mode at 70,000 resolution in the 60–900 m/z range, 4 kV spray voltage, 15 sheath gas and 5 auxiliary gas, operated in negative or positive ion mode (separate runs). These chromatographic and MS conditions were applied for both relative and targeted quantitative metabolomics measurements, with the differences that for the latter targeted quantitative post hoc analyses were performed on the basis of the stable isotope-labeled internal standards used as a reference quantitative measurement, as detailed below.

#### Lipidomics:

Samples were resolved as described,^4–6, 45^ over an ACQUITY HSS T3 column (2.1 × 150 mm, 1.8 μm particle size (Waters, MA, USA) using an aqueous phase (A) of 25% acetonitrile and 5 mM ammonium acetate and a mobile phase (B) of 50% isopropanol, 45% acetonitrile and 5 mM ammonium acetate. Samples were eluted from the column using either the solvent gradient: 0–1 min 25% B and 0.3 mL/min; 1–2 min 25–50% B and 0.3 mL/min, 2–8 min 50–90% B and 0.3 mL/min, 8–10 min 90–99% B and 0.3 mL/min, 10–14 min hold at 99% B and 0.3 mL/min, 14–14.1 min 99 – 25% B 1and 0.3 mL/min, 14.1–16.9 min hold at 25% B and 0.4 mL/min, 16.9–17 min hold at 25% B and resume flow of 0.3 mL/min. isocratic elution of 5% B flowed at 250 pl/min and 25°C or a gradient from 0–5% B over 0.5 min; 5–95% B over 0.6 min, hold at 95% B for 1.65 min; 95 – 5% B over 0.25 min; hold at 5% B for 2 min, flowed at 450 μl/min and 35°C^53^. The Q Exactive mass spectrometer (Thermo Fisher Scientific, San Jose, CA, USA) was operated independently in positive or negative ion mode, scanning in Full MS mode (2 pscans) from 150 to 1500 m/z at 70,000 resolution, with 4 kV spray voltage, 45 shealth gas, 15 auxiliary gas.

MS2 analyses for untargeted metabolomics For discovery mode untargeted metabolomics, dd-MS2 was performed at 17,500 resolution, AGC target = 1 e5, maximum IT = 50 ms, and stepped NCE of 25, 35 for positive mode, and 20, 24, and 28 for negative mode, as described in Stefeanoni et al. Haematologica 20 20,^77^ and applied to similar samples (i.e., stored RBCs) in D’Alessandro et al. Haematologica 20 2 0^57^.

### Quality control and data processing

Calibration was performed prior to analysis using the Pierce™ Positive and Negative Ion Calibration Solutions (Thermo Fisher Scientific). Acquired data was then converted from .raw to .mzXML file format using Mass Matrix (Cleveland, OH, USA). Samples were analyzed in randomized order with a technical mixture (generated by mixing 5 ul of all samples tested in this study) injected every 10 runs to qualify instrument performance. This technical mixture was also injected three times per polarity mode and analyzed with the parameters above, except CID fragmentation was included for unknown compound identification (10 ppm error for both positive and negative ion mode searches for intact mass, 50 ppm error tolerance for fragments in MS2 analyses – further details about the database searched below).

### Metabolite assignment and relative quantitation

Metabolite assignments, isotopologue distributions, and correction for expected natural abundances of deuterium, ^13^C, and ^15^N isotopes were performed using MAVEN (Princeton, NJ, USA),^78^ against an in house library of deuterated lipid standards (SPLASH® LIPIDOMIX® Mass Spec Standard, Avanti Lipids) and in house libraries of 3,000 unlabeled (MSMLS, IROATech, Bolton, MA, USA; IroaTech ; product A2574 by ApexBio; standard compounds for central carbon and nitrogen pathways from SIGMA Aldrich, St Louis, MO, USA) and labeled standards (see below for the latter). Discovery mode analysis was performed with standard workflows using Compound Discoverer 2.1 SP1 (Thermo Fisher Scientific, San Jose, CA). From these analyses, metabolite IDs or unique chemical formulae were determined from high-resolution accurate intact mass, isotopic patterns, identification of eventual adducts (e.g., Na + or K+, etc.) and MS^2^ fragmentation spectra against the KEGG pathway, HMDB, ChEBI, and ChEMBL databases. Additional untargeted lipidomics analyses were performed with the software LipidSearch (Thermo Fisher, Bremen, Germany).

### Simultaneous thrombin and plasmin generation assay (STPGA)

Simultaneous evaluation of thrombin and plasmin generation (TG and PG, respectively) was performed as described previously^79^. Briefly, plasma samples were mixed with either thrombin specific substrate, Z-Gly-Gly-Arg-AMC (Bachem, Bubendorf, Switzerland) or plasmin specific substrate, Boc-Glu-Lys-Lys-AMC (Bubendorf, Switzerland) and 16 nM of thrombomodulin (PeproTech, Rocky Hill, NJ, USA). The reaction was initiated by adding an activator solution that yielded a final concentration of 1 μM tissue factor (Diagnostica Stago, Parsippany, NJ, USA), 0.7 Mg/mL of tissue plasminogen activator (Sigma-Aldrich, St. Louis, MO, USA) and 16 mM CaCl2. Sample wells supplemented with buffer (150 mM NaCl and 20 mM HEPES) and AMC fluorophore instead of activator solution were used for background and calibrator measurements respectively. Calculation of thrombin and plasmin concentration was performed as described previously ^80^.

#### VWF, FVIII and ADAMTS13 activity and antigen quantitation:

The antigen and activity measurement of VWF and ADAMTS13 was performed by using commercial ELISA kits. VWF antigen and collagen binding activity levels were measured by using Human Von Willebrand Factor ELISA Kit (ab168548, Abcam, Cambridge, UK) and TECHNOZYM® vWF:CBA ELISA Kit (5450301, Technoclone, Vienna, Austria) respectively. ADAMTS13 antigen and activity levels were measured by using Human ADAMTS13 ELISA Kit (ab234559, Abcam) and TECHNOZYM® ADAMTS13 Activity ELISA (5450701, Technoclone) respectively. FVIII antigen levels were measured by using Human Factor VIII total antigen assay ELISA kit (HFVNIKTTOT, Molecular Innovations, Novi, MI, USA). All assays were performed following manufacturer’s recommendations with additional dilution of plasma samples as required.

### Statistical Analysis

Graphs and statistical analyses (either t-test or repeated measures ANOVA) were prepared with GraphPad Prism 5.0 (GraphPad Software, Inc, La Jolla, CA), GENE E (Broad Institute, Cambridge, MA, USA), and MetaboAnalyst 4.0.^81^ In MetaboAnalyst, relative quant data (but not for abs quant), raw values for integrated peak areas for each metabolite were normalized on a pool of day 0 controls and auto-scaled for each species independently prior to margining all the data for multivariate analysis. Analyses through MetaboAnalyst included principal component analysis, partial least square discriminant analysis, hierarchical clustering analyses (including time-series repeated measures and two-way ANOVA analyses), calculation of receiver operating characteristic (ROC) curves, correlation analyses (Spearman) and machine learning analyses (random forest, support-vector machine – SVM).

## Supplementary Material

Supplement 1

Supplement 2

## Key Points

- Patient age, sex, and body mass index have significant effects on clinical and metabolic markers of thromboinflammatory, cardiovascular and renal dysfunction, as well as prognosis, in hospitalized patients with and without COVID-19;
- The primary metabolic pathways affected include tryptophan/kynurenine metabolism, arginine/proline/creatinine metabolism and fatty acids/acyl-carnitines;
- Clinical history and pre-existing conditions significantly affect metabolic processes in these pathways and may influence prognosis.
