# Supplementary material for: Biological and Clinical Factors contributing to the Metabolic Heterogeneity of Hospitalized Patients with and without COVID-19
Source: Res Sq. 2021 May 10:rs.3.rs-480167. Preprint. [Version 1] doi: 10.21203/rs.3.rs-480167/v1 (PMC8132252; doi:10.21203/rs.3.rs-480167/v1)
Supplement: Supplement 2 [file c42b2c4ec5c866697522b629.pdf]

SUPPLEMENTARY MATERIAL

TABLE OF CONTENTS

SUPPLEMENTARY FIGURES 2

SUPPLEMENTARY FIGURE 1 2
SUPPLEMENTARY FIGURE 2 3
SUPPLEMENTARY FIGURE 3 5
SUPPLEMENTARY FIGURE 4 6
SUPPLEMENTARY FIGURE 5 8
SUPPLEMENTARY FIGURE 6 9
SUPPLEMENTARY FIGURE 7 11
SUPPLEMENTARY FIGURE 8 12
SUPPLEMENTARY FIGURE 9 13
SUPPLEMENTARY FIGURE 10 14
SUPPLEMENTARY FIGURE 11 15
SUPPLEMENTARY FIGURE 12 16
SUPPLEMENTARY FIGURE 13 17
SUPPLEMENTARY FIGURE 14 18
SUPPLEMENTARY FIGURE 15 19
SUPPLEMENTARY FIGURE 16 20

SUPPLEMENTARY DATA TABLE *(PLEAE REFER TO “LEGEND” SHEET WITHIN THE FILE)* .... **XLSX**

SUPPLEMENTARY FIGURES

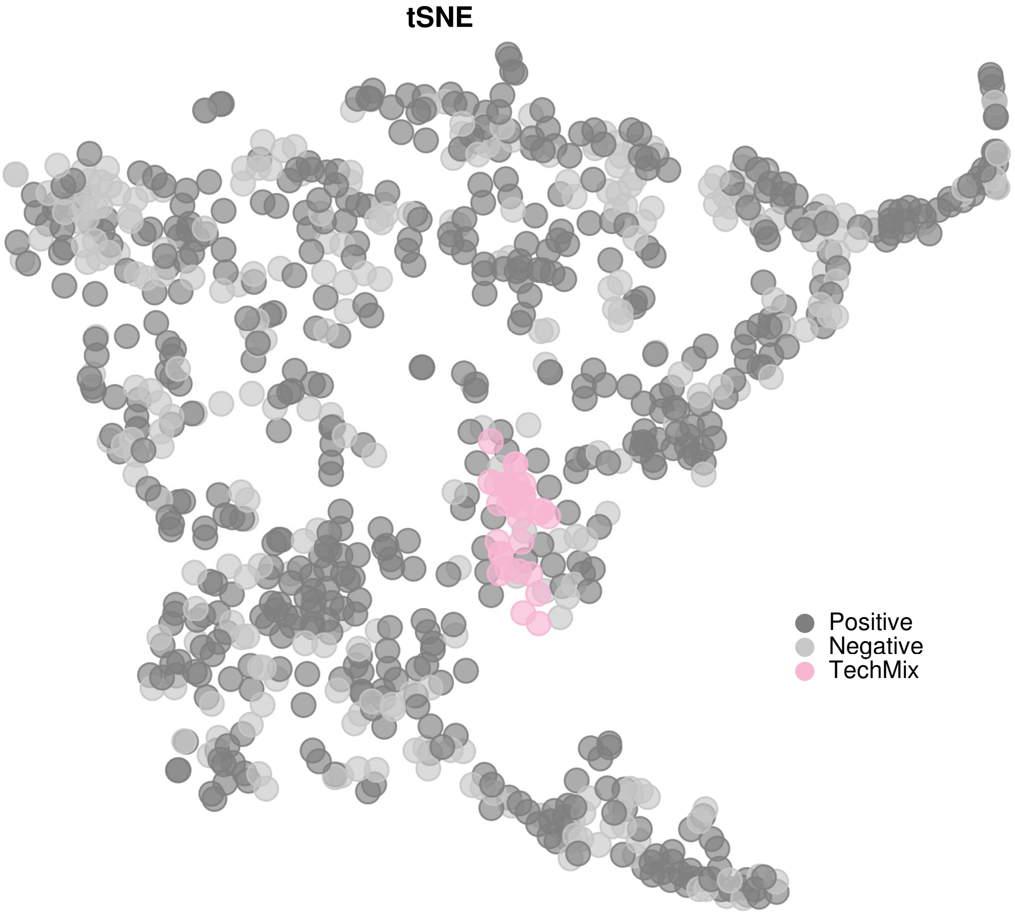

Supplementary Figure 1 – tSNE Analysis of Metabolomics data confirms quality of the analysis, with technical mixes clustering together (pink).

Impact of IL-6

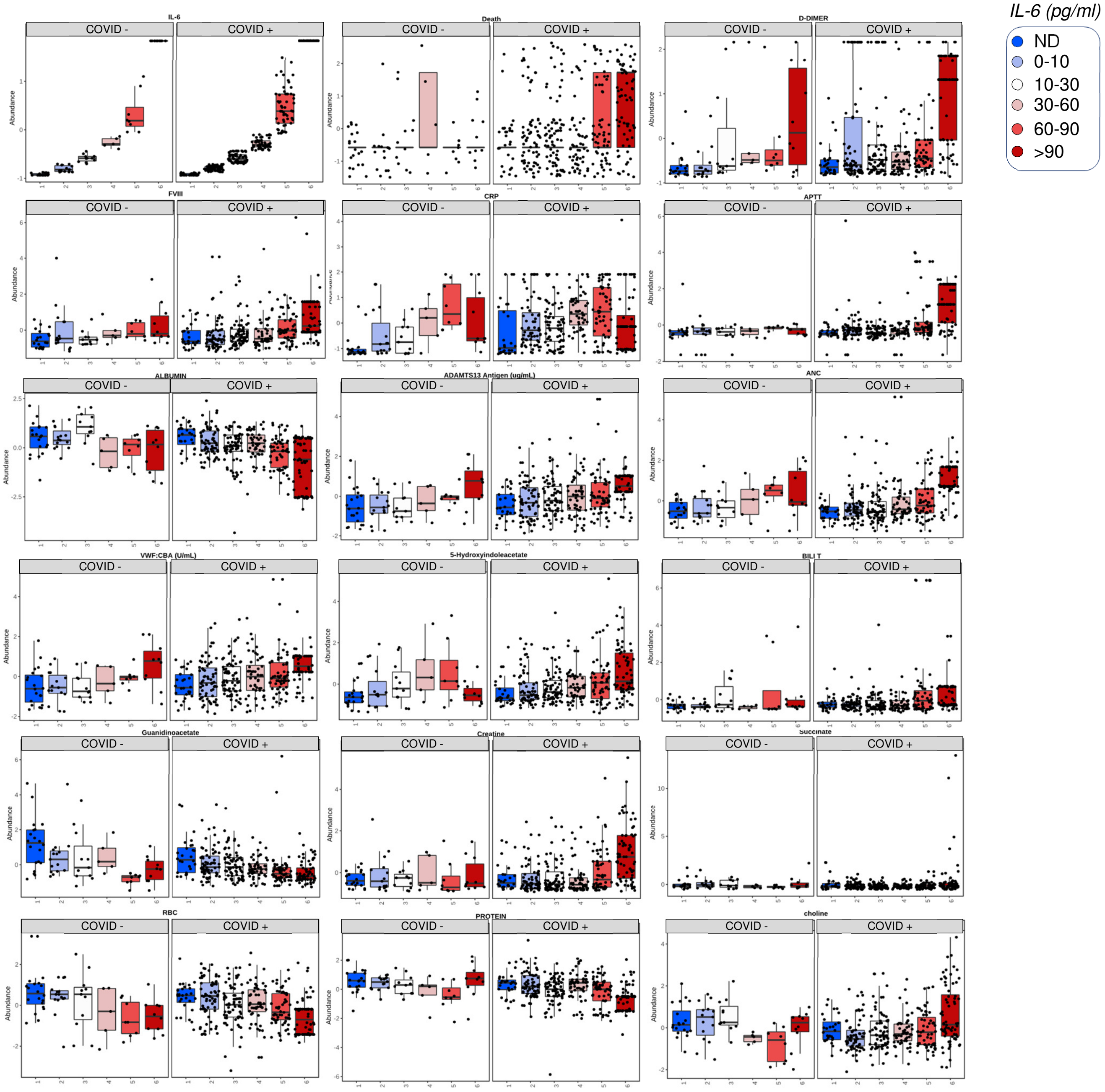

continues →

Impact of IL-6

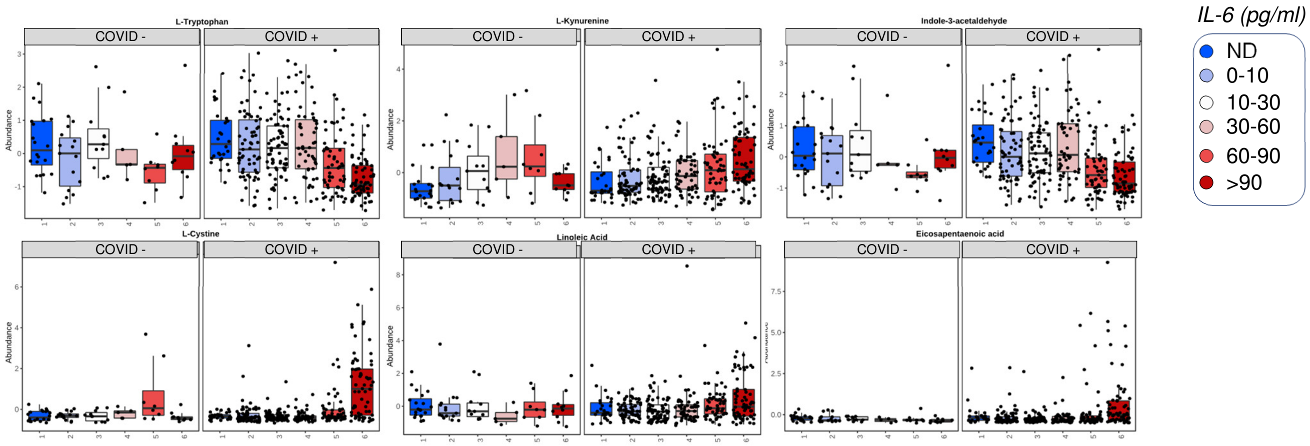

Supplementary Figure 2 – Markers of blood group in COVID negative (-) and positive (+) patients, as determined by Two-way ANOVA.

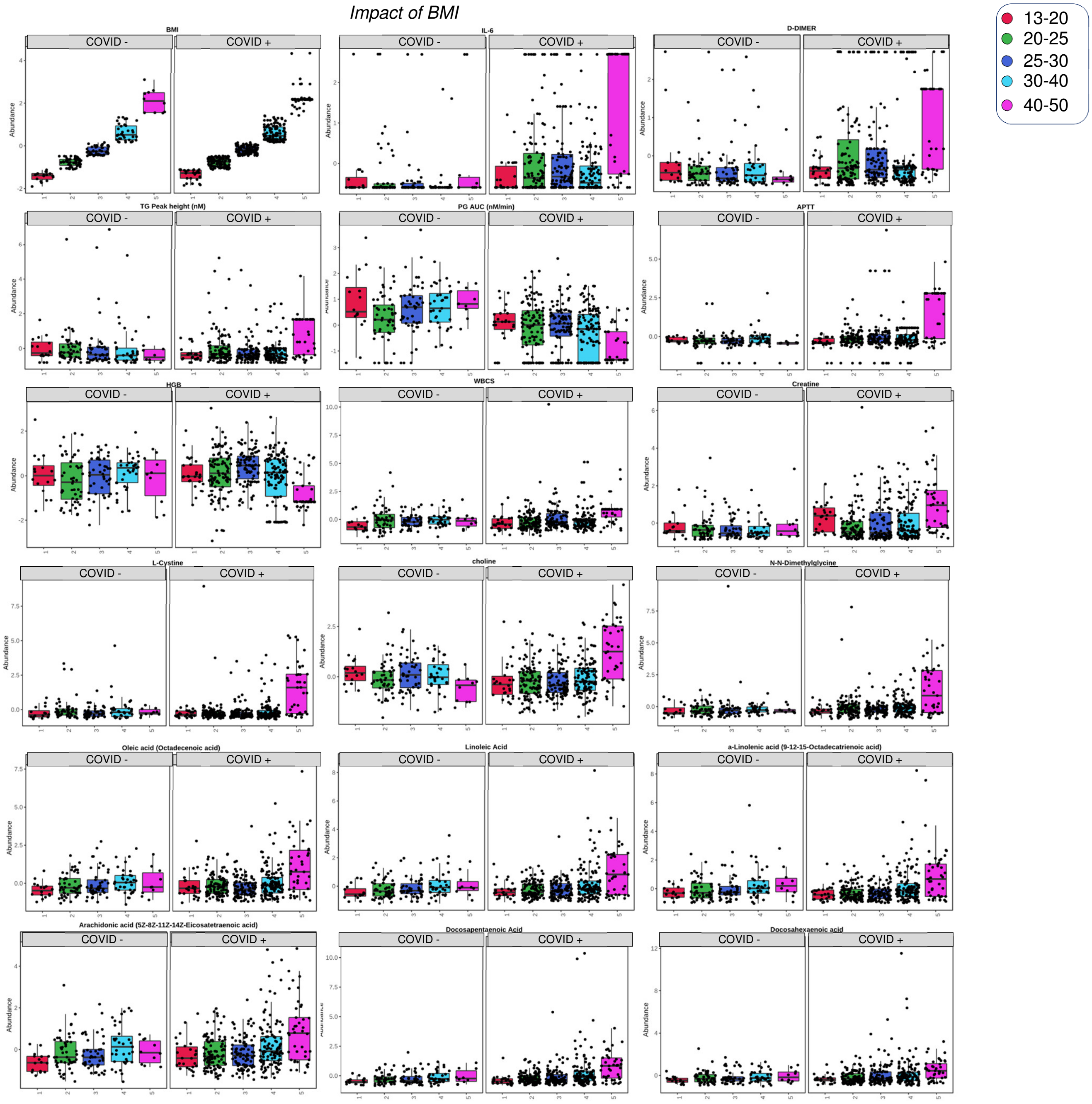

Supplementary Figure 3 – Markers of BMI in COVID negative (-) and positive (+) patients, as determined by Two-way ANOVA.

Impact of Age

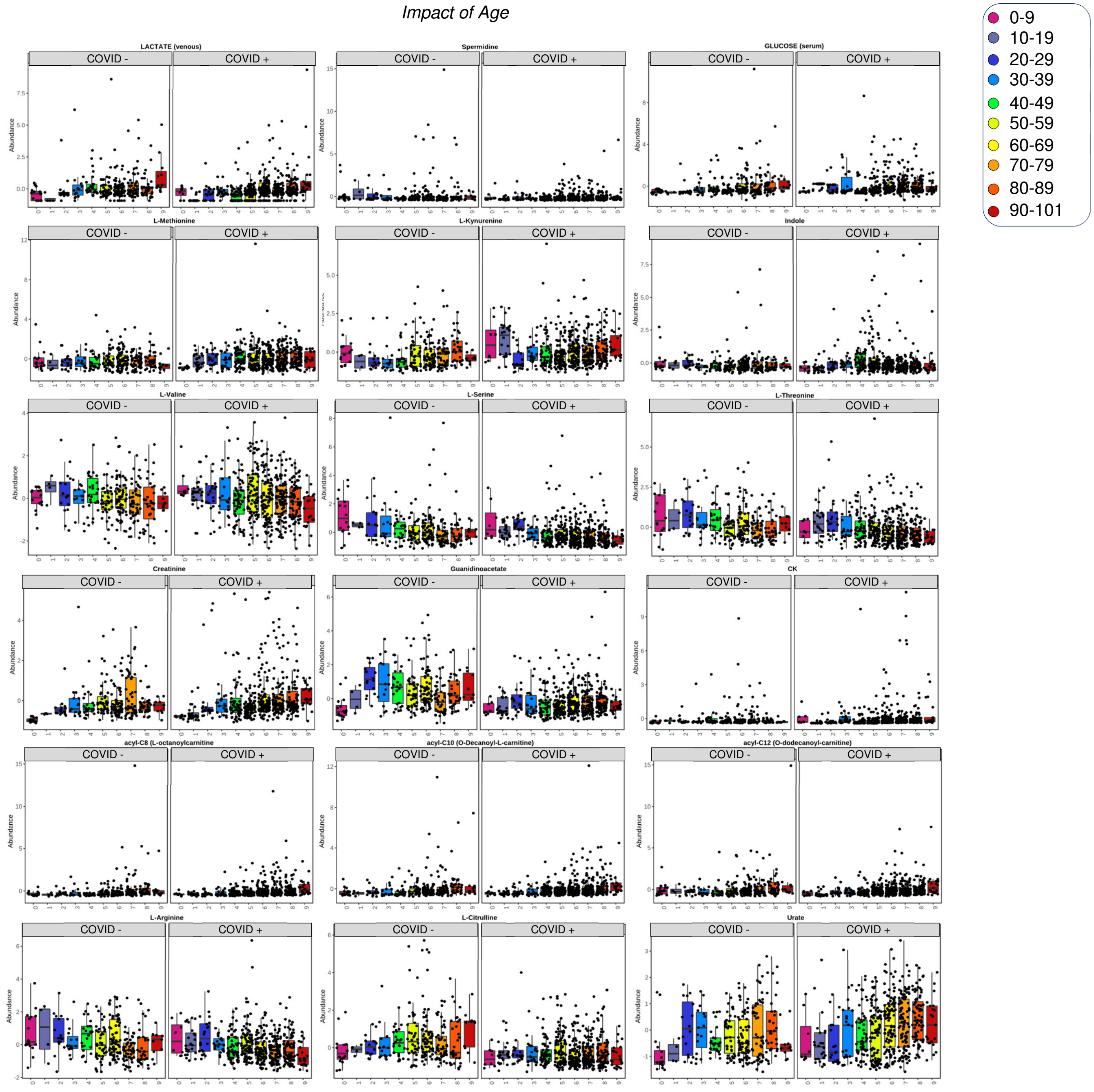

continues →

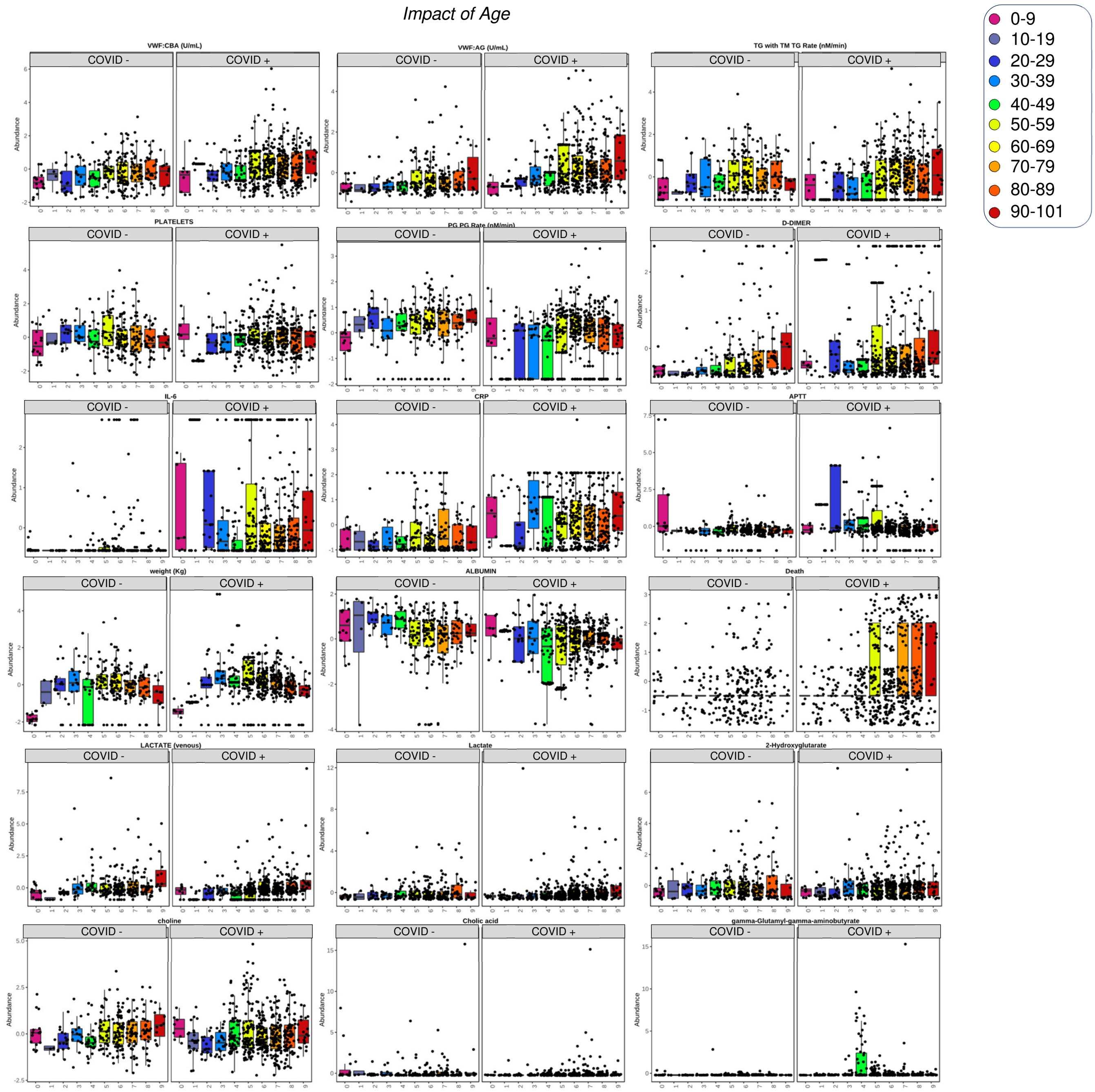

Supplementary Figure 4 – Markers of age in COVID negative (-) and positive (+) patients, as determined by Two-way ANOVA.

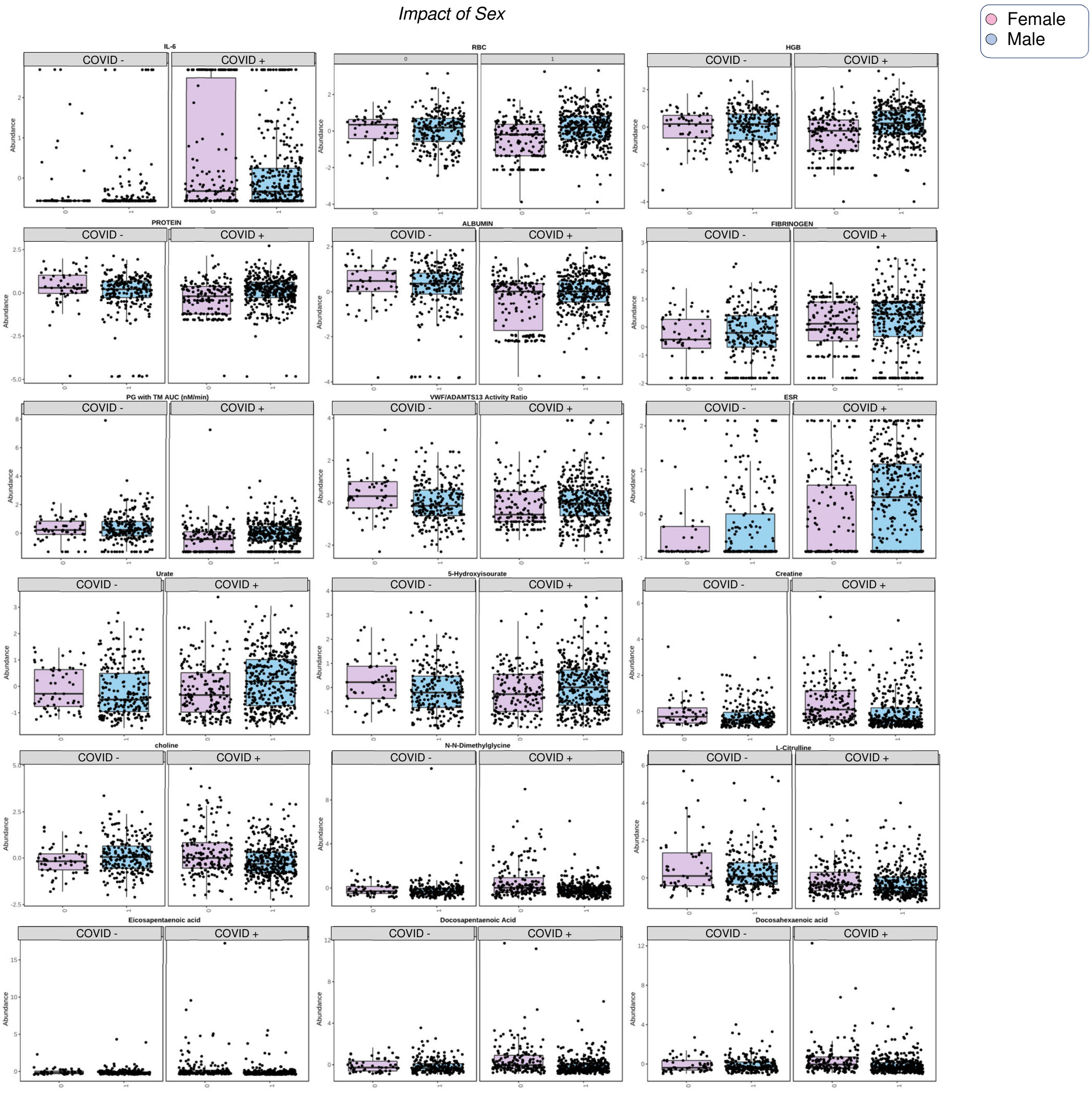

Supplementary Figure 5 – Markers of sex in COVID negative (-) and positive (+) patients, as determined by Two-way ANOVA (0 = female; 1 = male).

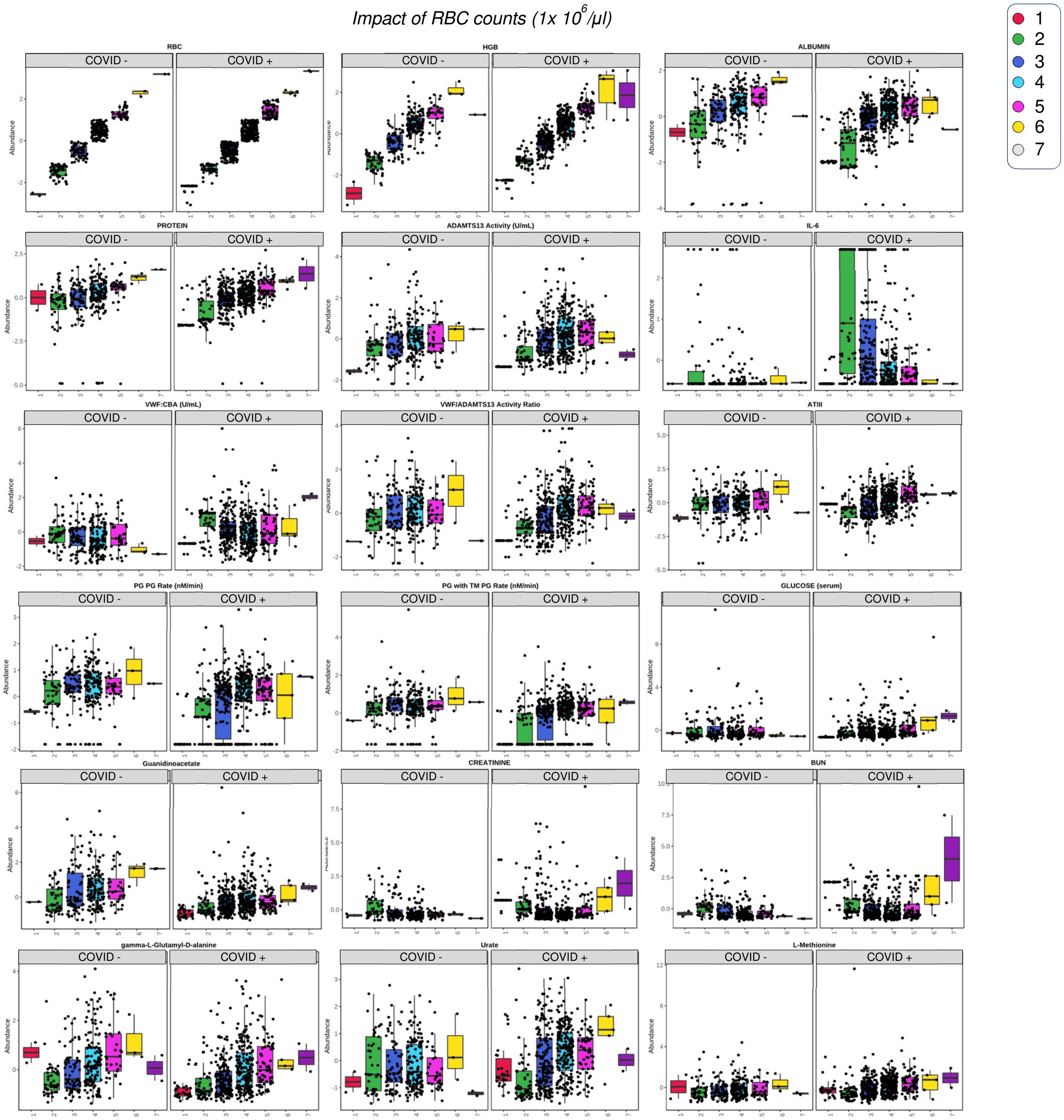
Impact of RBC counts (1x 10^6/µl)
RBC
HGB
ALBUMIN
PROTEIN
ADAMTS13 Activity (U/mL)
IL-6
VWF:CBA (U/mL)
VWF/ADAMTS13 Activity Ratio
ATIII
PG PG Rate (nM/min)
PG with TM PG Rate (nM/min)
GLUCOSE (serum)
Guanidinoacetate
CREATININE
BUN
gamma-L-Glutamyl-D-alanine
Urate
L-Methionine
COVID -
COVID +
Abundance
1
2
3
4
5
6
7

continues →

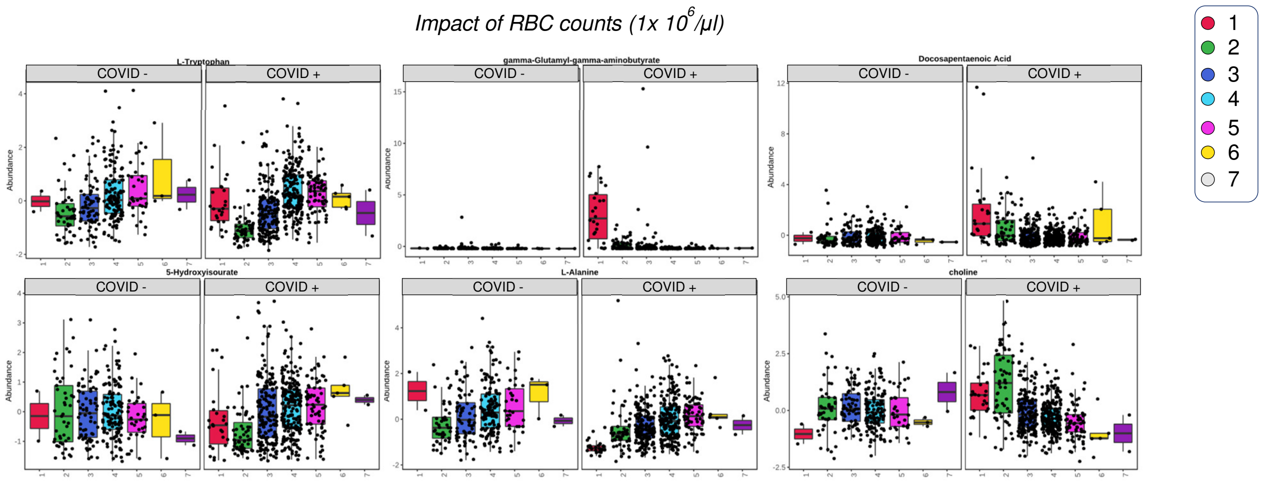

Supplementary Figure 6 – Markers of RBC counts in COVID negative (-) and positive (+) patients, as determined by Two-way ANOVA (numbers in the legend indicate RBC count in millions per microliter).

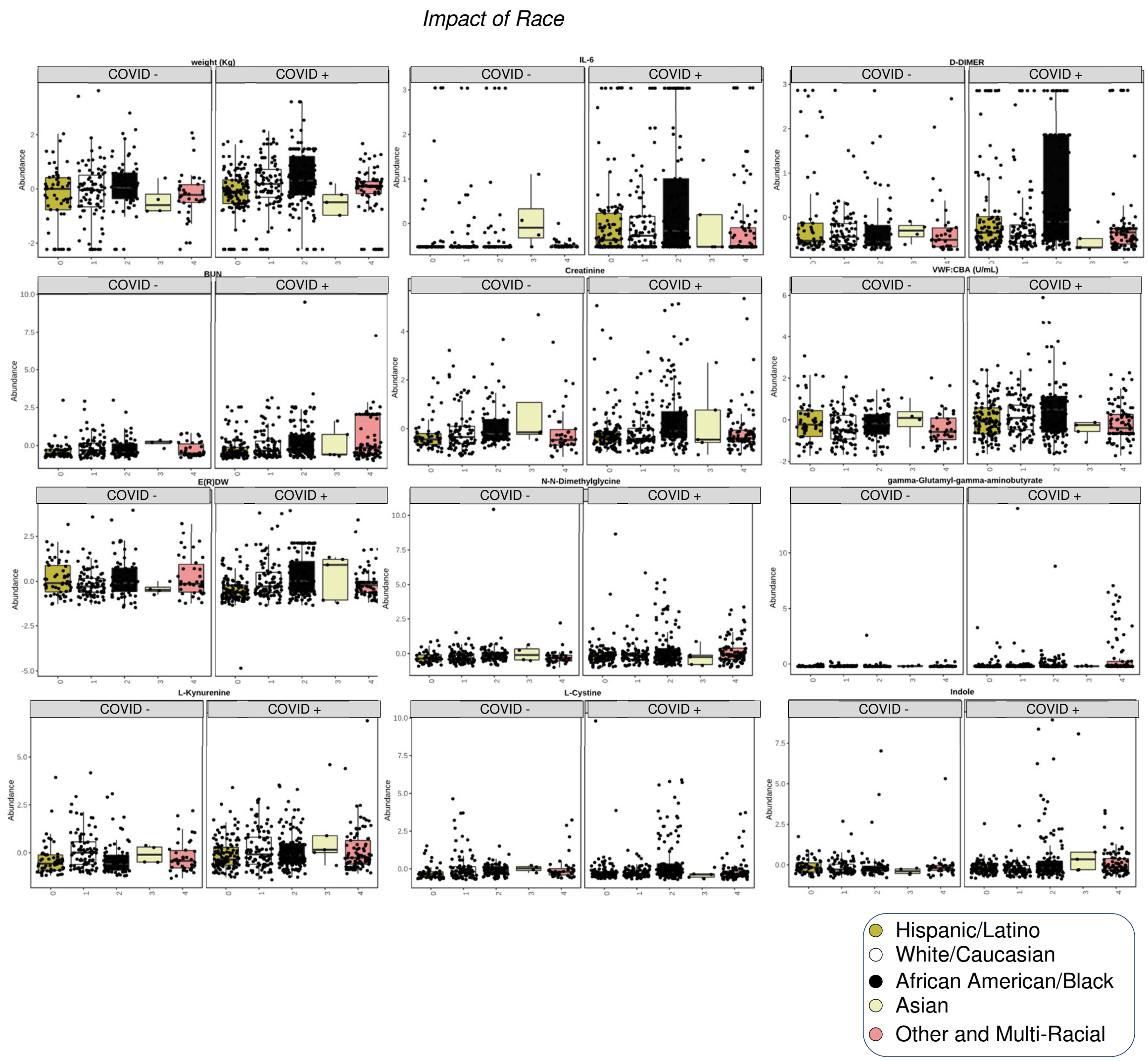

Supplementary Figure 7 – Markers of race in COVID negative (-) and positive (+) patients, as determined by Two-way ANOVA.

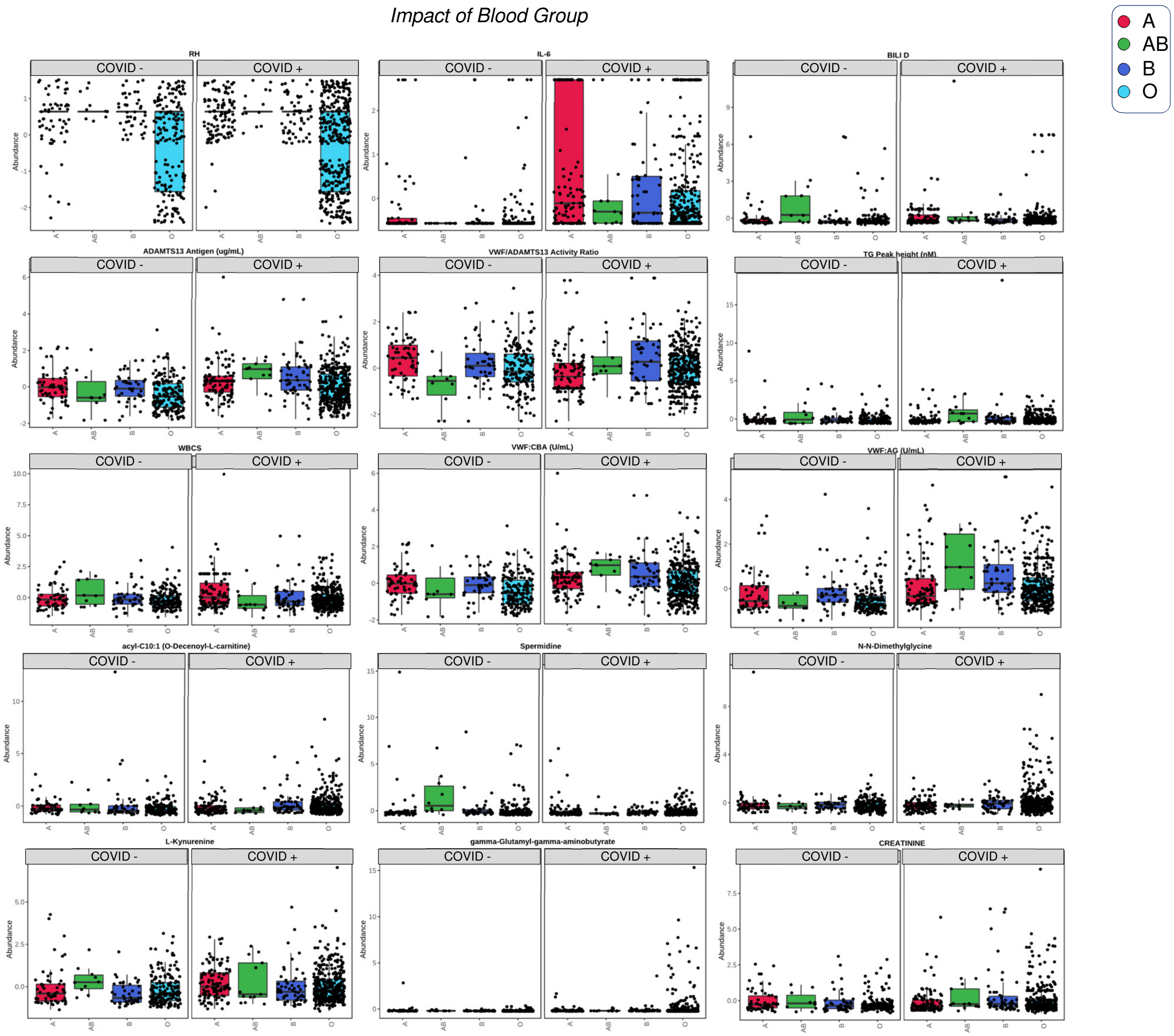

Supplementary Figure 8 – Markers of blood group in COVID negative (-) and positive (+) patients, as determined by Two-way ANOVA.

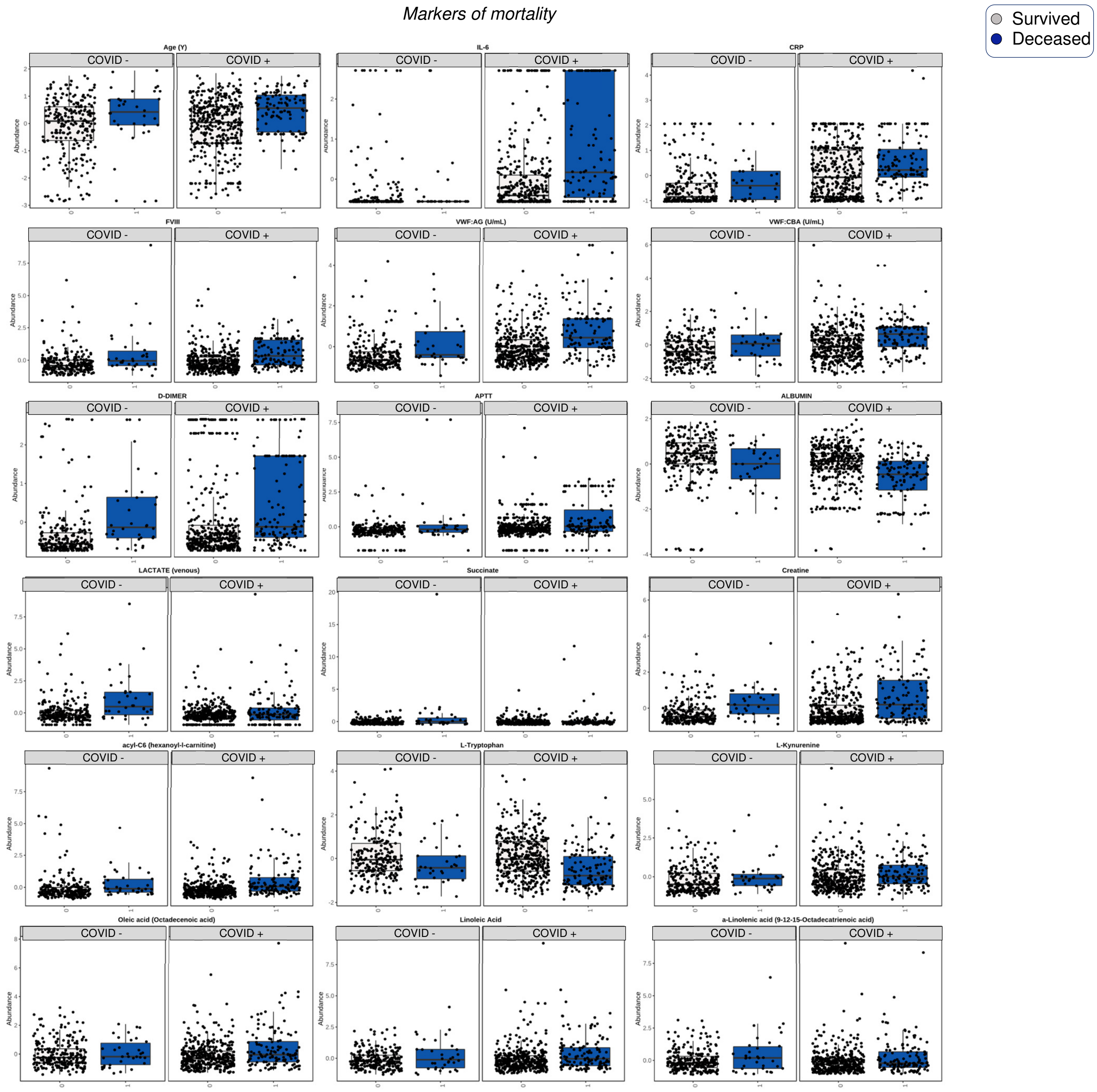

Supplementary Figure 9 – Markers of mortality in COVID negative (-) and positive (+) patients, as determined by Two-way ANOVA (0 = survived; 1 = dead).

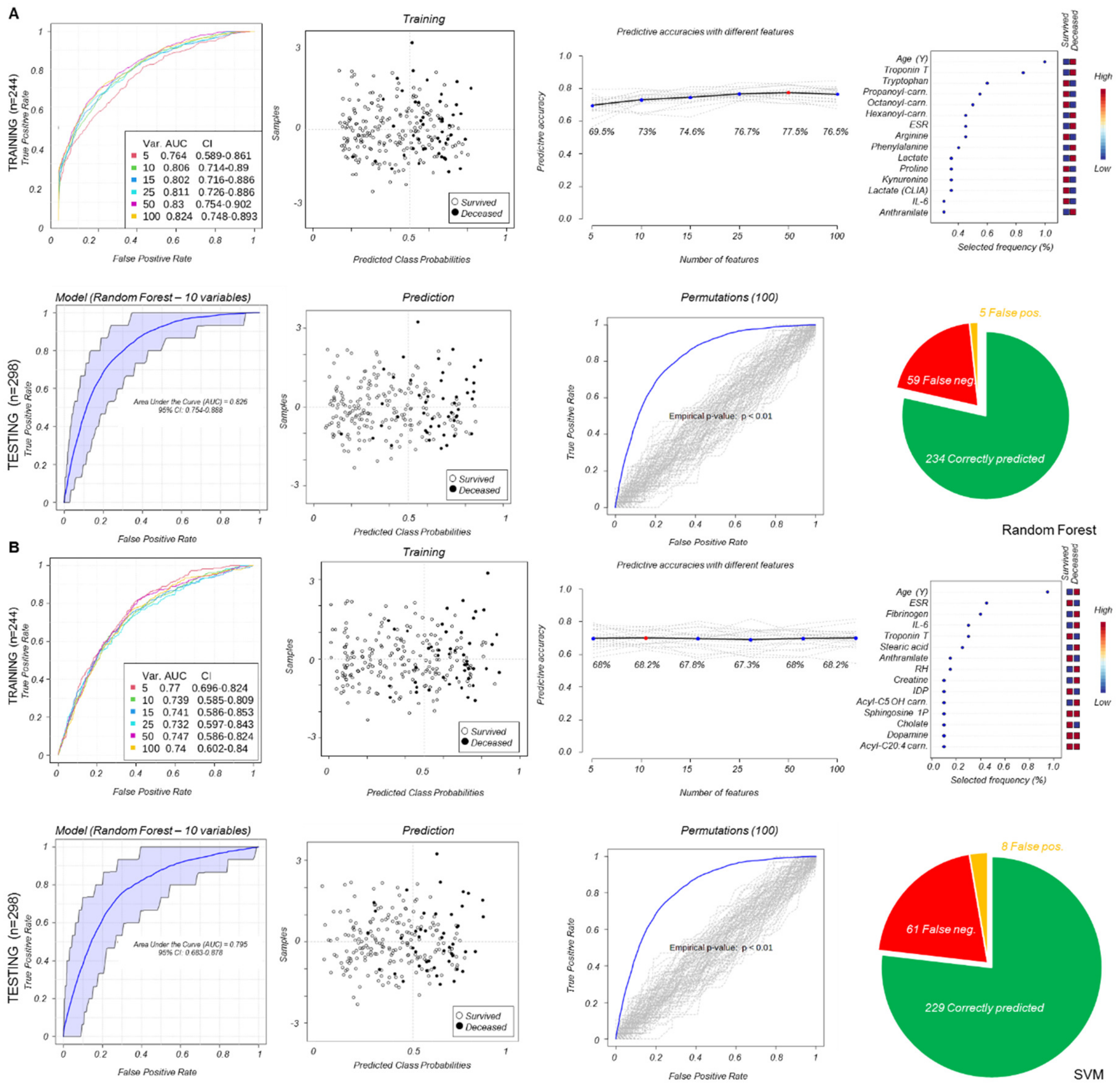

Supplementary Figure 10– Comparison of predictive models of mortality via Random Forest (A) and SVM (B) algorithms. Patients were curated for metabolomics, clinical, coagulation and inflammatory variables (total n = 542). The cohort was then divided in two groups, one for training (n = 244) and one for testing (n = 298) of the algorithm, which resulted to be ~78% and ~75% accurate, respectively.

Impact of being on ventilator

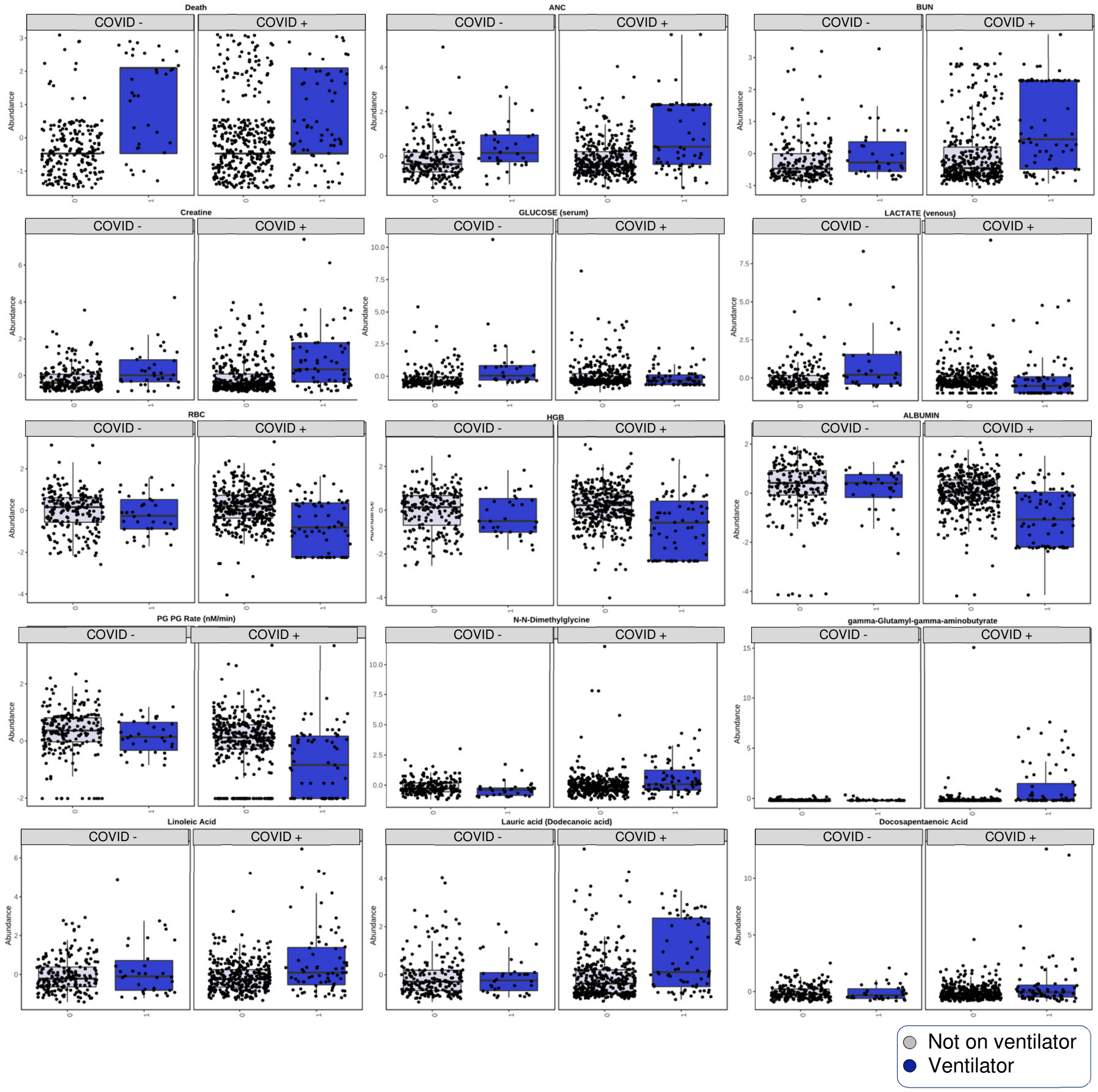

Supplementary Figure 11 – Markers of ventilators in COVID negative (-) and positive (+) patients, as determined by Two-way ANOVA (0 = not on ventilator; 1 = on ventilator).

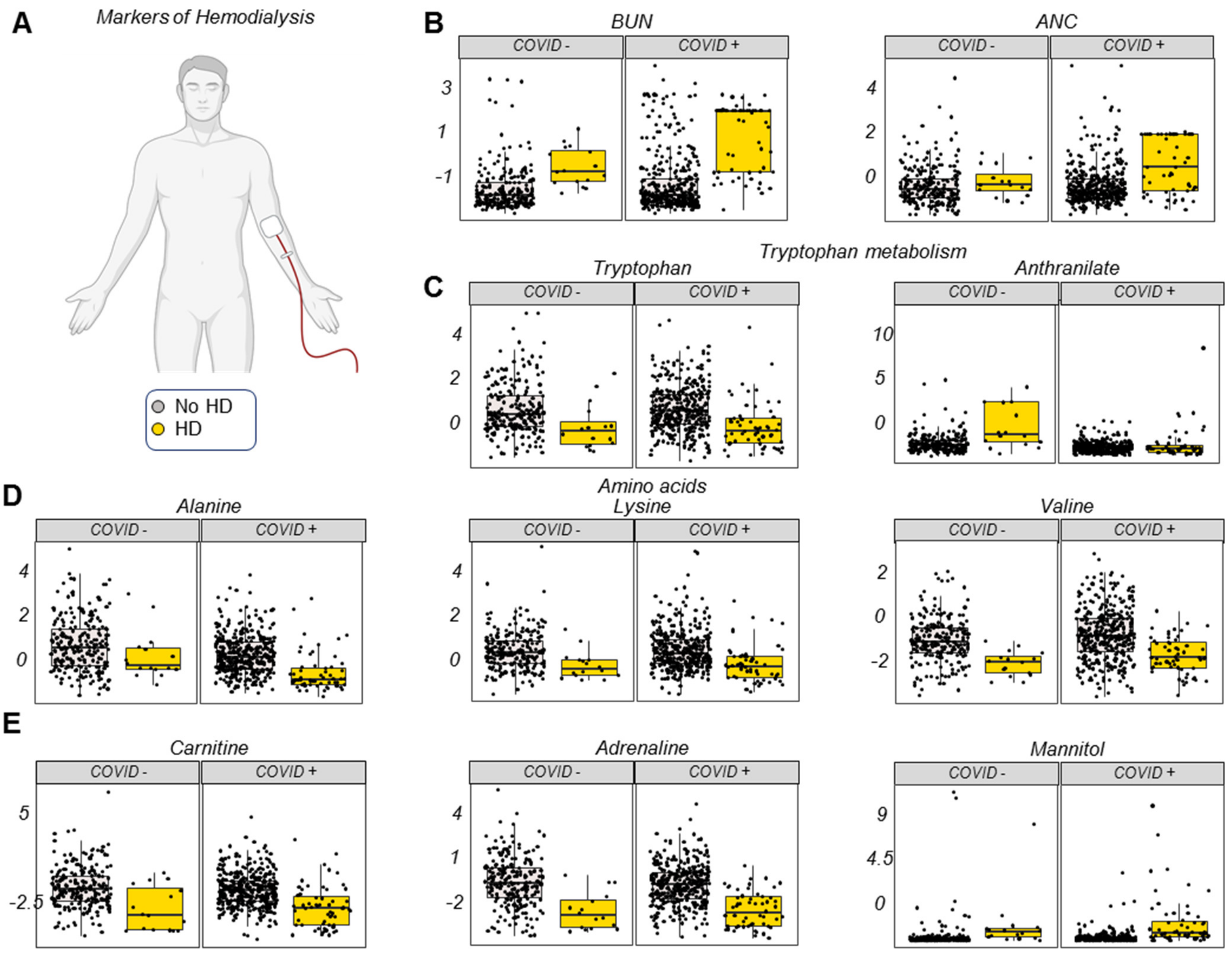

Supplementary Figure 12 – Markers of hemodialysis (without clotting) in COVID negative (-) and positive (+) patients, as determined by Two-way ANOVA (0 = no hemodialysis; 1 = on hemodialysis).

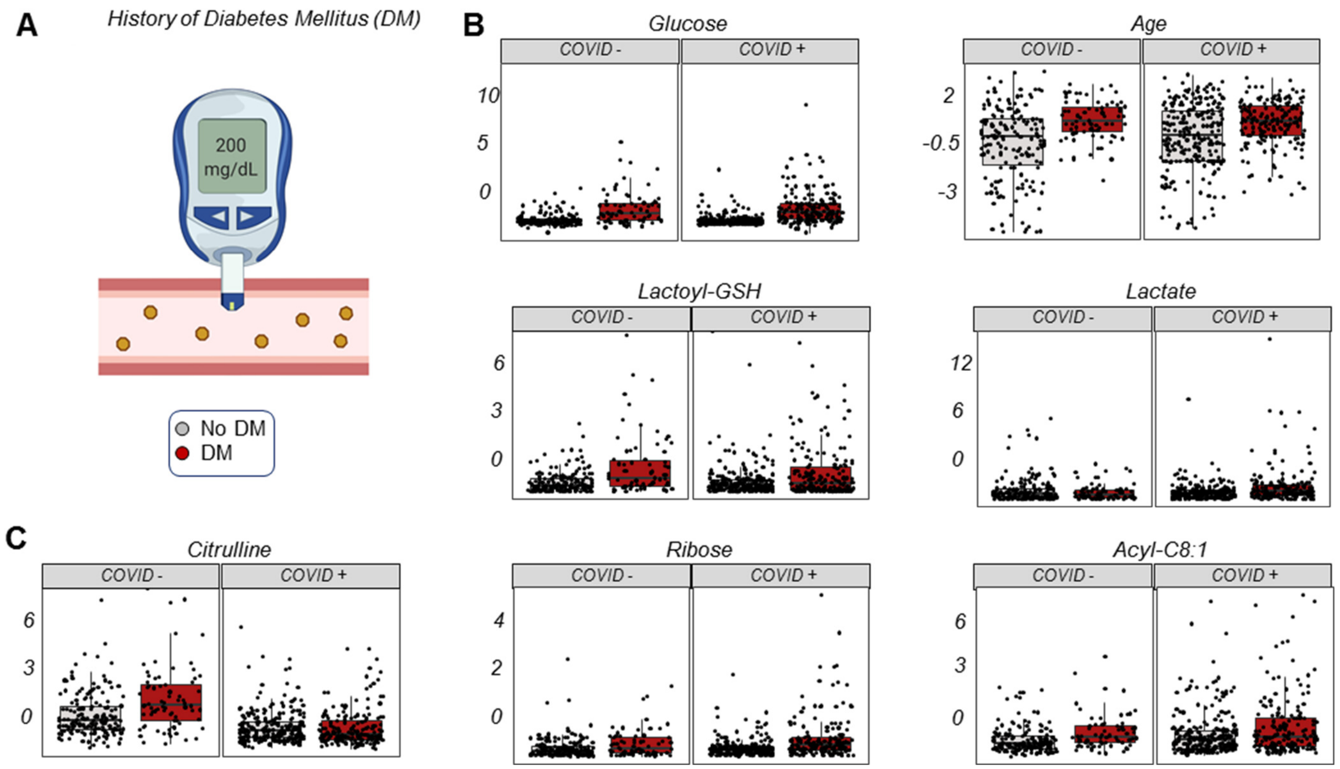

Supplementary Figure 13 – Impact of pre-existing conditions such as diabetes in COVID negative (-) and positive (+) patients, as determined by Two-way ANOVA (0 = no history of diabetes; 1 = diabetes).

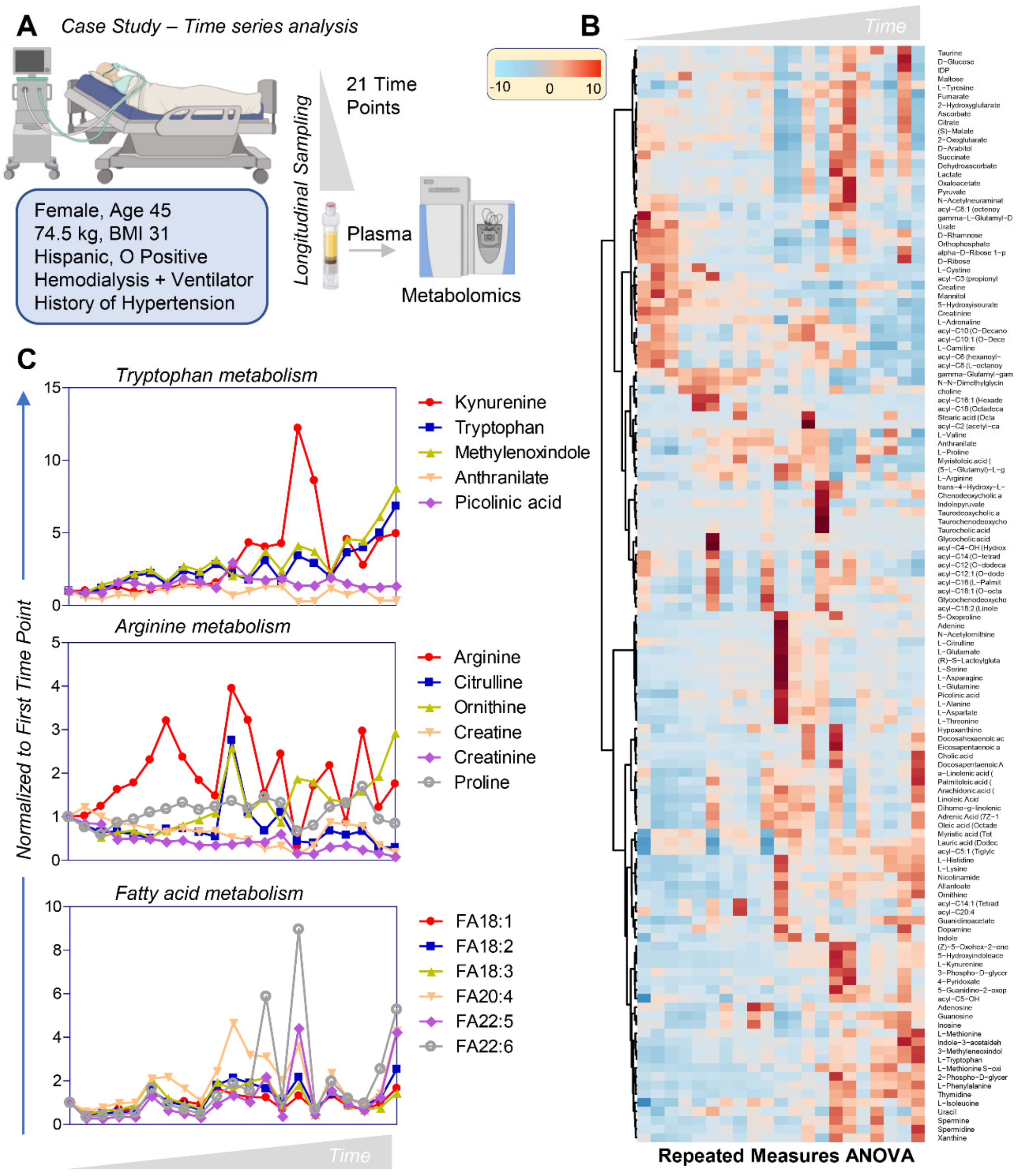

Supplementary Figure 14 – Time course analysis of a critically ill patient who survived.

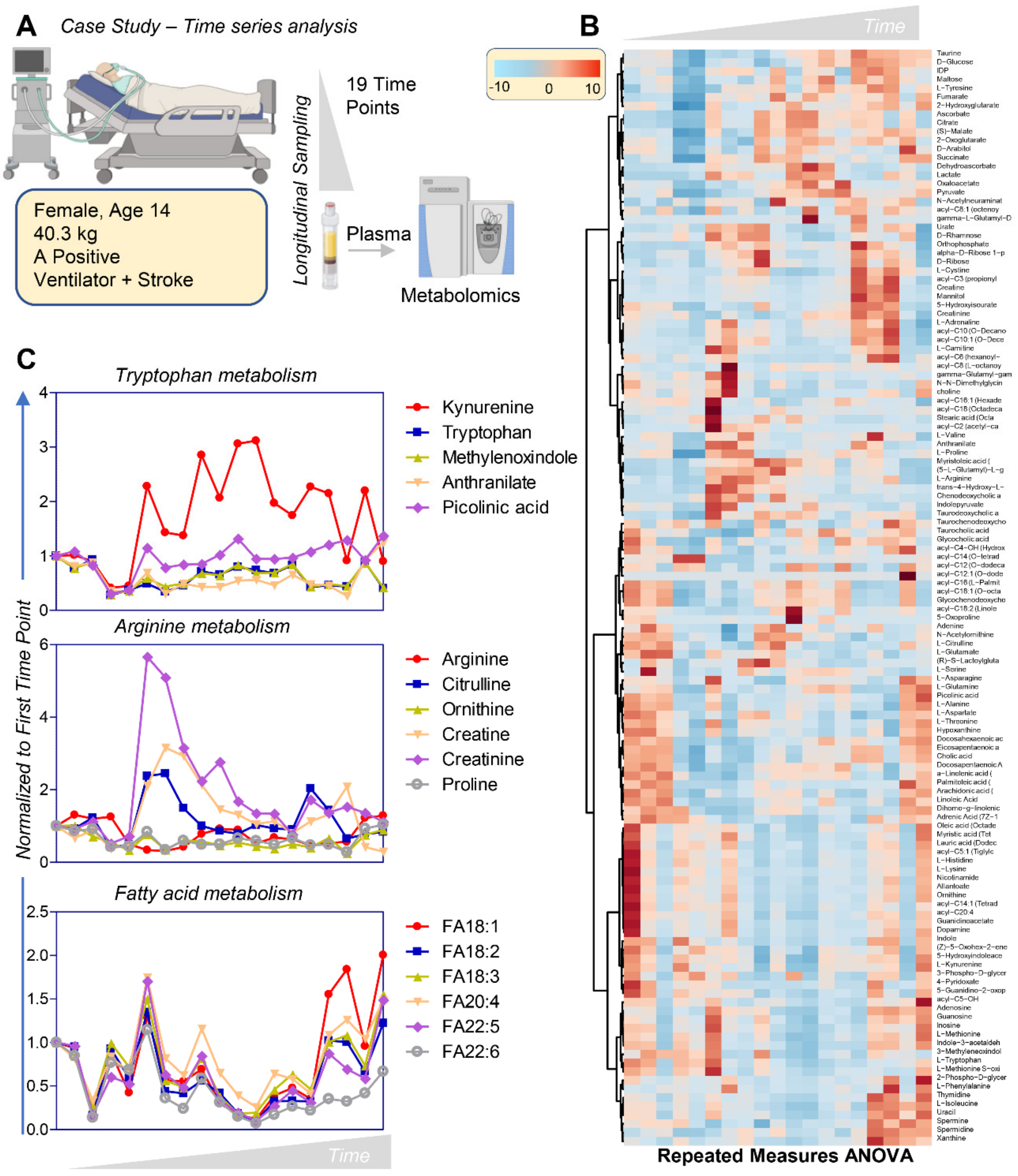

Supplementary Figure 15 – Time course analysis of a critically ill patient who survived (vectorial version of in manucript figure).

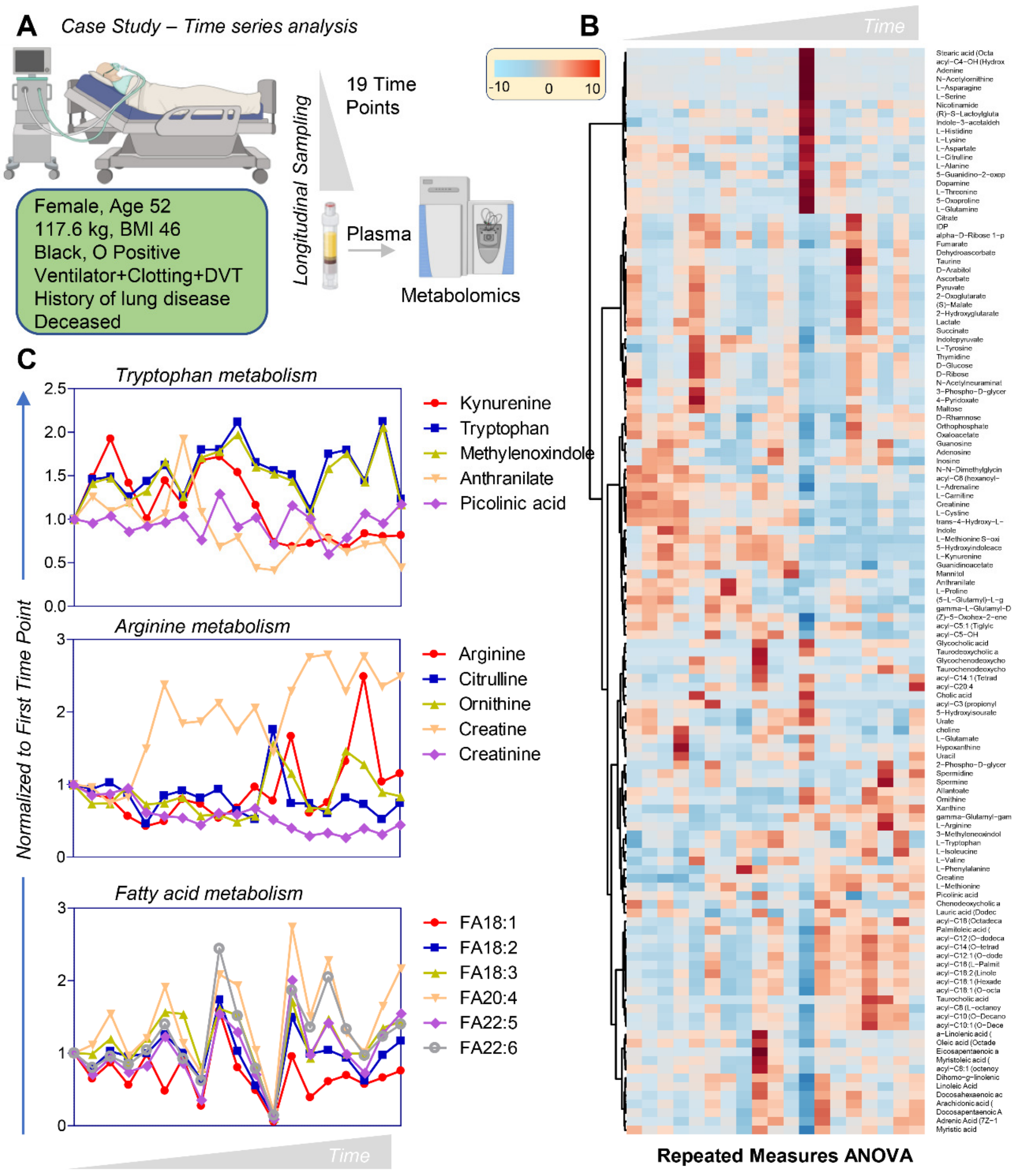

Supplementary Figure 16 – Time course analysis of a critically ill patient who died (vectorial version of in manucript figure).
